# Whole genome pool sequencing reveals genomic variation in field *Haemonchus contortus* isolates in Sweden

**DOI:** 10.1186/s12864-026-13449-5

**Published:** 2026-10-17

**Authors:** Nanxing Liu, Paulius Baltrušis, Åsa Ohlsson, Johan Höglund, Sofia Mikko

**Affiliations:** Department of Animal Biosciences, Swedish University of Agricultural Sciences, Uppsala, Sweden

**Keywords:** Pool-seq, Nematode, Population genomics, Genomic variation

## Abstract

**Background:**

*Haemonchus contortus* is a major gastrointestinal parasite of small ruminants worldwide. Its large effective population size, high fecundity, and extensive genetic variation promote rapid adaptation and the development of anthelmintic resistance. Resolving genome-wide genetic variation in *H. contortus* within and among populations is essential for tracking variant distribution, interpreting resistance-associated markers, and informing parasite control strategies. Although reduced representation and targeted marker approaches have provided important insights into population structure, whole genome sequencing is required to fully characterise population differentiation. This study aimed to characterise genome-wide variation within and between field-derived *H. contortus* populations in Sweden, using whole genome sequencing of pools of individuals from 12 sheep farms.

**Results:**

Field-derived *H. contortus* isolates from Swedish farms showed high genomic diversity within populations (nucleotide diversity (Pi) = 0.0211–0.0253) and relatively low genomic differentiation among most populations (fixation index (F_ST_) < 0.1). However, three population pairs displayed higher genomic differentiation (F_ST_ = 0.144–0.157) combined with lower genomic nucleotide diversity (Pi = 0.0211–0.0227), with divergence concentrated on chromosomes 4, 5, and X. Significantly differentiated single nucleotide polymorphisms were linked to a limited set of annotated genes involved in regulatory, signalling, and nervous system-related functions. However, the biological basis of these patterns remains unclear and will require further investigation using phenotype-linked data and farm-management metadata.

**Conclusions:**

This work establishes the first genome-wide baseline for field-derived *H. contortus* populations across multiple Swedish sheep farms and shows that pooled-sample whole-genome sequencing (Pool-seq) is a useful method for describing broad population-genomic patterns in this species. Our findings indicate relatively low genomic variation between Swedish *H. contortus* populations and high genomic variation within them. This study provides a valuable foundation for future research on parasite adaptation, resistance, surveillance, and control.

**Supplementary information:**

The online version contains supplementary material available at https://doi.org/10.1186/s12864-026-13449-5.

## Background

The abomasal nematode *Haemonchus contortus* is one of the most prevalent and economically significant parasites affecting livestock worldwide. Although it originated in sub-Saharan Africa, *H. contortus* has spread globally, including temperate regions [1–3]. It infects a broad range of domestic and wild artiodactyl hosts [4], with sheep and goats being the most severely affected [5]. *H. contortus* is notable for its remarkable reproductive capacity, producing up to ten thousand eggs daily [6], which enables infections to build up rapidly in grazing animals. By attaching to the abomasal mucosa and extracting blood, the subadult and adult worms cause severe pathological effects, including anaemia, oedema, and, in severe cases, mortality [7]. These health impacts impair animal productivity and necessitate substantial investment in treatment, resulting in considerable annual losses to the livestock industry [8, 9]. These characteristics, together with its history of rapidly evolving drug resistance, make *H. contortus* not only a major veterinary pathogen but also a powerful model for studying how genetic variation drives parasite adaptation across hosts, environments and control practices [10–12].

At the population level, the genomic structure of *H. contortus* is shaped by processes occurring both at the parasite-host interface and in the environment. The high fecundity and short generation time of *H. contortus*, combined with faecal-oral transmission on pastures, provide ample opportunity for extensive genetic variation. This genetic variation is further influenced by both anthropogenic and natural forces, including strong selection imposed by anthelmintic treatment, transmission‑related population bottlenecks shaped by management practices, and climate‑driven environmental selection [11–13]. Patterns of population genetic structure were first described using *H. contortus* mitochondrial DNA (mtDNA) in 1995 [14], providing a framework for interpreting parasite population structure in terms of gene flow, genetic drift, and selection, as well as for assessing the extent to which local populations are demographically and evolutionarily independent units. Subsequent studies have shown that *H. contortus* harbours extraordinarily high levels of genetic diversity, with most of this variation occurring within populations rather than among them [12, 15–17]. The high levels of genetic variation in *H. contortus* populations together with its prolificity and short life span, certainly contribute to its remarkable adaptive capacity and rapid development of resistance to multiple anthelmintic drug classes, posing a major global challenge for parasite control [4, 6, 11, 18]. Understanding this genetic variation within and among populations is therefore essential for tracking genome-wide population dynamics, identifying selective sweeps associated with key traits, such as anthelmintic resistance, and ultimately developing genetic tools for surveillance of sustainable parasite control strategies [4, 8, 15].

Population structure and genetic diversity in *H. contortus* and other helminths have been investigated using various molecular approaches, including microsatellite markers [19, 20], targeted Single Nucleotide Polymorphism (SNP) panels [21], Restriction Site-Associated DNA Sequencing (RAD-seq) [22–24], exome sequencing [22, 25], and amplicon sequencing [8, 9, 13, 15, 26–30]. Although these reduced representation and targeted methods have provided valuable insights, they capture only a small and potentially biased fraction of the genome, limiting inference of genome-wide evolutionary processes. In recent years, parasite genomics has increasingly shifted towards whole genome sequencing (WGS), driven by the increased availability of high-throughput sequencing technologies and significant reductions in sequencing costs [22, 31]. This shift has enabled both single-worm and pooled-sample WGS (Pool-seq), which together offer complete, genome-wide resolution and allow more comprehensive population genetic and genomic analyses. Single-worm WGS requires extensive resources and is often constrained by the difficulty of isolating and sequencing large numbers of individuals, especially from field samples or early life stage parasites with low genomic DNA yield [22, 32, 33]. Pool-seq addresses these limitations by sequencing DNA from many individuals together, providing a cost-effective approach for estimating genome-wide allele frequencies and comparing populations across a wide range of eukaryotic taxa [34–36]. It is particularly well suited to helminths such as *H. contortus*, where large census population sizes, difficulty accessing adult worms, and limited DNA yield restrict individual sequencing.

Despite recent technological advances, population genomic studies of *H. contortus* based on WGS remain relatively limited, particularly for naturally infected populations, compared with marker-based studies. To address this gap, we used Pool-seq to analyse field-derived *H. contortus* isolates collected from multiple sheep farms in Sweden, representing the first study of its kind. This study aimed to characterise the genomic landscape and patterns of genetic variation within and among populations at both genome-wide and chromosome-wide scales, and to assess the utility of Pool-seq for reliably capturing broad population genomic patterns in field-derived nematode populations. The study establishes a genome-wide baseline for Swedish *H. contortus* populations and offers a resource for future surveillance of anthelmintic resistance markers and for comparing Swedish populations with other European and/or global *H. contortus* populations.

## Methods

### Sample collection and DNA extraction

**Fig. 1:**
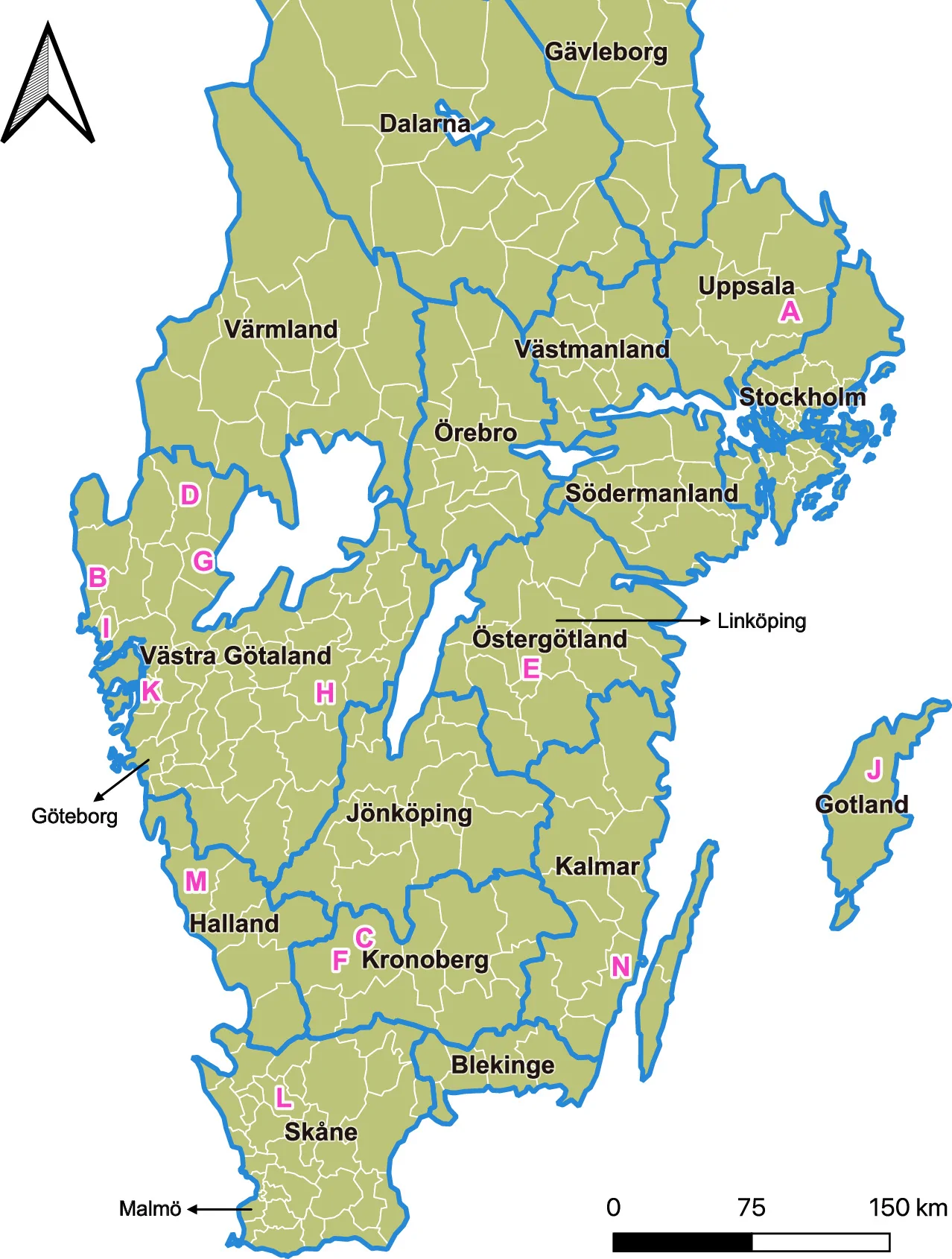
A map illustrating the geographical origins of the various farm populations (A-N) in Sweden. For confidentiality reasons, the locations shown are approximate and do not represent the exact farm locations. Samples J and M were excluded from downstream analyses due to poor mapping quality

Fourteen sheep farms across Sweden were sampled, each farm representing a distinct field population of *H. contortus* (Fig. 1). Eleven farms contributed with one sample each, while three farms (A, F, and G) contributed two biological replicate samples each, yielding a total of 17 faecal samples (Table 1). Each sample consisted of composite faeces collected on one occasion from three randomly selected sheep per farm, with equal contributions from each sheep. Biological replicate samples were collected from two different groups of three sheep in the same flock and farm. The inclusion criteria for samples were a high egg count (>500 EPG) and a high proportion of *H. contortus* (>80%). Both egg counting and morphological identification were conducted using microscopy at a local diagnostic laboratory (Vidilab AB, Enköping, Sweden). All faecal samples were cultured simultaneously under optimal conditions to obtain infective third-stage larvae of *H. contortus*, following a previously used protocol [3]. For each sample, a comparable packed larval volume was prepared, comprising a pool of at least 2,000 larvae.

**Table 1:** Geographical origins for the different farm population samples

Sample ID	Farm population	Counties of Sweden
A1^a^	A	Uppland
A2^a^	A	Uppland
B	B	Västra Götaland
C	C	Kronoberg
D	D	Västra Götaland
E	E	Östergötland
F1^a^	F	Skåne
F2^a^	F	Skåne
G1^a^	G	Västra Götaland
G2^a^	G	Västra Götaland
H	H	Västra Götaland
I	I	Västra Götaland
J	J	Gotland
K	K	Västragötaland
L	L	Skåne
M	M	Halland
N	N	Småland

^a^Sample A1 and A2 are biological replicates from farm A population; sample F1 and F2 are biological replicates from farm F population; sample G1 and G2 are biological replicates from farm G population. All other samples are from distinct farms

High molecular weight genomic DNA was extracted from each larval pool using the QIAsymphony SP instrument and the QIAsymphony DSP DNA Mini Kit (Qiagen, Hilden, Germany), following the manufacturer’s tissue pretreatment and purification protocol (Tissue_LC_200_V7_DSP) with the following modifications: i) samples were incubated in 1.8 mg/ml RNase A and 220 ul Buffer ATL (Qiagen, Hilden, Germany) prior to initiating the procedure; and ii) samples were homogenised at 6000 rpm for 2 × 20 s, using Precellys CK14 beads and a Precellys Evolution Touch Homogenizer (Bertin Corp., Rockville, MD, USA) before proteinase K treatment, followed by overnight incubation at 56 °C. DNA quantity and quality were assessed using a TapeStation 4150 system with Genomic DNA ScreenTape (Agilent, Santa Clara, CA, USA), a NanoDrop 8000 spectrophotometer (Thermo Fisher Scientific, Waltham, MA, USA), and a Qubit 2.0 fluorometer with the Qubit dsDNA HS assay Kit (Thermo Fisher Scientific, Waltham, MA, USA).

### Whole genome sequencing

Genomic DNA from the 17 larval pools was submitted to the SNP&SEQ Technology Platform (National Genomics Infrastructure Sweden and Science for Life Laboratory, Uppsala, Sweden) for WGS. Barcoded sequencing libraries were prepared from 100 ng DNA per sample using the Illumina TruSeq Nano DNA Library Prep Kit (Illumina Inc., San Diego, CA, USA). Paired-end sequencing (150 bp) was performed on an Illumina NovaSeq X Plus platform using XLEAP-SBS sequencing chemistry, with all 17 libraries loaded onto a single lane of a 10B flow cell. Raw sequence data were delivered as compressed FASTQ files.

### Data processing and downstream analyses

**Fig. 2:**
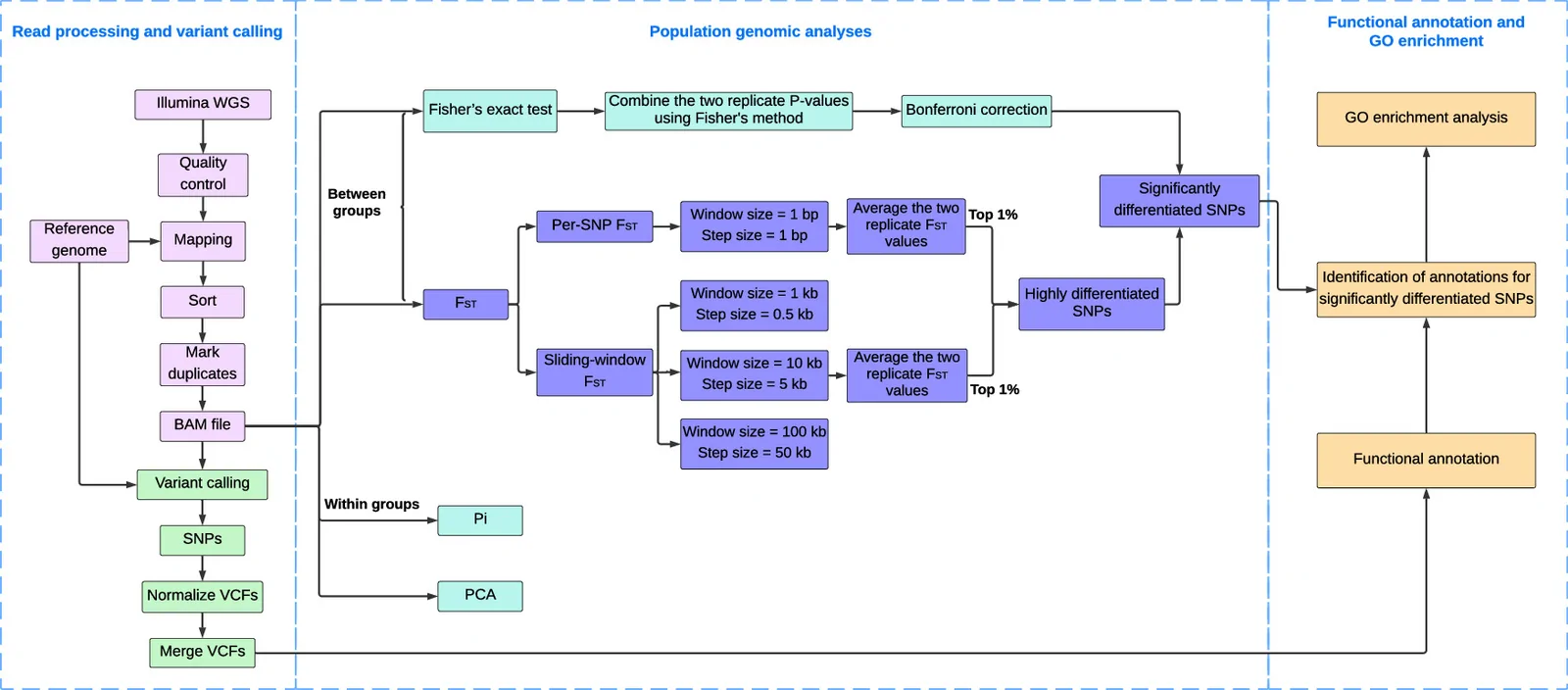
Bioinformatics analysis workflow. Overview of the analytical pipeline used in this study, from read processing and variant calling to population genomic analyses using F_ST_, Pi, and PCA, followed by functional annotation and GO enrichment analysis. Colours are used to distinguish the major stages of the workflow

Initial data processing was conducted using Linux command-line tools and Bash scripts, while downstream analyses, statistical testing, and visualisation were performed in Python v3.12.4 and R v4.4.1 [37]. All code supporting this study is available on GitHub (https://github.com/nxl365/hcontortus-pooled-wgs_2025.git). The analytical workflow is summarised in Fig. 2.

### Mapping and variant calling

Raw FASTQ files were assessed using FastQC v0.12.1 (Babraham Bioinformatics, Cambridge, UK) and summarised with MultiQC v1.22.3 [38]. Reads were trimmed with fastp v0.24.0 [39] (fastp -g -c -q 20 -l 20) to remove adapters and low-quality bases. Cleaned reads were aligned to the *H. contortus* reference genome [16] using BWA*-*MEM v0.7.18 [40]. Resulting SAM files were converted to coordinate-sorted BAM files using SAMtools sort v1.20 [41]. Mapping statistics and coverage were obtained using SAMtools flagstat and SAMtools coverage. Duplicate reads were marked using Picard v3.3.0 (https://broadinstitute.github.io/picard). Samples J and M were excluded from further analyses due to poor mapping quality. Subsequent analyses were therefore conducted on the remaining 15 samples (representing 12 farms).

Variant calling was performed using FreeBayes v1.3.7 [42] (freebayes -m 20 -q 30 --pooled-continuous) with duplicate-marked BAM files as input. All other parameters were kept at their default settings, including the minimum alternate allele fraction (-F) of 0.05. SNPs were extracted and filtered for quality and depth using BCFtools v1.20 (bcftools view -v snps; bcftools filter -i ‘QUAL > = 20 & INFO/DP > = 10’). Filtered variants were retained in Variant Call Format (VCF) for downstream analyses.

### Genomic differentiation between farms quantified by fixation index (F_ST_)

All population genomics analyses were conducted using only the nuclear genome. A multi-sample pileup file was generated from the 15 BAM files using SAMtools mpileup (samtools mpileup -d 500 --min-MQ 20 --min-BQ 30) and converted to a synchronised file using Popoolation2 v1.201 [43, 44] (popoolation2 mpileup2sync.jar --min-qual 20 --fastq-type sanger). The synchronised file, containing allele frequency estimates for each sample at every genomic position across the reference genome, was subsequently used to analyse pairwise genomic differentiation using Popoolation2. Differentiation was quantified as the fixation index (F_ST_) and evaluated using Fisher’s exact test (FET), as previously described [10, 43, 45, 46].

Pairwise sliding-window F_ST_ analysis was performed using three window sizes (1 kb, 10 kb, 100 kb) with corresponding step sizes (0.5 kb, 5 kb, 50 kb) (popoolation2 fst-sliding.pl --suppress-noninformative --min-count 4 --min-coverage 15 --max-coverage 2% --pool-size 2000 --window-size 1000/10000/100000 --step-size 500/5000/50000). To compare the three window-size settings, the total number of windows and the mean number of SNPs per window were calculated. For each farm population pair, the interquartile range (IQR) of F_ST_ values across all genomic windows was first calculated, and then the median of these pair-specific IQRs was used to summarise F_ST_ variability for each window size.

Genome-wide and chromosome-specific pairwise F_ST_ values were summarised as weighted means across windows, with weights defined as the number of SNPs per window. Within-farm consistency was assessed by comparing biological replicates, whereas between-farm differentiation was analysed across all 12 farms. For farms represented by two biological replicates, mean F_ST_ values across replicates were used in downstream analyses. The three most divergent farm population pairs (those with the highest genome-wide pairwise F_ST_ values) were selected for further investigation. Ten-kilobase windows within the top 1% of the genome-wide sliding-window F_ST_ distribution were defined as highly differentiated windows.

Per-SNP F_ST_ was calculated to identify highly differentiated SNPs between farms (for farms represented by two biological replicates, mean F_ST_ values across replicates were used) (popoolation2 fst-sliding.pl --suppress-noninformative --min-count 4 --min-coverage 15 --max-coverage 2% --pool-size 2000 --window-size 1 --step-size 1). For each of the three most divergent farm population pairs, highly differentiated SNPs were retained if they: i) ranked within the top 1% of the genome-wide per-SNP F_ST_ distribution, and ii) were located within highly differentiated windows identified as described above.

To detect statistically significant differences in allele frequencies between farm populations while accounting for variation in sequencing depth, FET was applied to each SNP for all pairwise population comparisons [43] (popoolation2 fisher-test.pl --suppress-noninformative --min-count 4 --min-coverage 15 --max-coverage 2%). For farms represented by two biological replicates, replicate P-values were combined using Fisher’s method [47] to obtain a single P-value per comparison. To control for Type I error, Bonferroni correction was applied (α_Bonf_ = 0.05/*m*, where *m* represents the total number of genome-wide SNPs tested in each comparison) for each pairwise comparison as previously described [10]. Among the retained highly differentiated SNPs, those with FET P-values lower α_Bonf_ were considered statistically significant [46].

### Principal component analysis to reveal population structure

The rc files, which include the major and minor alleles for each SNP across all samples, were generated from the previous synchronised file using Popoolation2 (popoolation2 snp-frequency-diff.pl --min-count 4 --min-coverage 15 --max-coverage 2%). A subset of biallelic SNPs was extracted by retaining only sites with parameters “deletion_sum = 0” and “allele_count = 2”. Genome-wide SNPs with a minor allele frequency greater than or equal to 0.05 in at least one sample were used for a principal component analysis (PCA) to summarise allele frequency covariation among samples. The first three principal components were visualised in three dimensions. The SNP subset was generated using custom code available on GitHub.

### Genomic diversity within farms quantified by nucleotide diversity (Pi)

To estimate within-farm genomic diversity, the multi-sample pileup file was first split into individual pileup files for each sample and chromosome. These files were then used as input for NPStat v1 [48] to calculate nucleotide diversity (Pi) in 10 kb non-overlapping windows (npstat -n 4000 -l 10000 -maxcov 90 -mincov 15 -minqual 20 -nolowfreq 4). Windows with fewer than 500 bases covered by reads (5% of the window size) were excluded from downstream analyses to reduce potential bias arising from missing data [49, 50]. Genome-wide and chromosome-wide Pi values were summarised as weighted means across windows, with weights defined as the number of bases covered by reads in each window. For farms represented by two biological replicates, the average Pi values across replicates were used.

The distribution of window-based Pi values was also analysed for each sample. Medians and IQRs were used to describe the distributions. For statistical comparisons, window-based Pi value distributions were first assessed for non-normality using the Shapiro–Wilk test. Differences among samples were then tested using the Kruskal–Wallis test. In cases where the Pi value comparisons showed significant divergence, pairwise differences between farms were evaluated using the Wilcoxon Rank Sum test [51]. For farms represented by two biological replicates, P-values were combined using the geometric mean. The resulting P-values were adjusted for multiple testing using the Benjamini–Hochberg correction.

### Functional annotation and gene ontology enrichment analysis

For each of the three most divergent farm pairs, the corresponding SNP VCF files from the two farms were processed and merged. Each VCF file was normalised, and multiallelic records were removed using BCFtools (bcftools norm -m-both | bcftools norm --check-ref w -f; bcftools view -v snps). The processed VCF files were then merged (bcftools merge -m none), resulting in files containing both overlapping and sample-specific SNPs. For the farm represented by two biological replicates, replicate VCF files were first processed and merged as described above prior to merging the two farms. Each merged VCF file was functionally annotated using SnpEff v5.2 [52] (snpEff snpEff.jar -ud 2000) with a custom database constructed from the GFF3 annotation file (WormBase release 2021-04) [53] and the *H. contortus* reference genome. In a separate analysis, each SNP VCF file from 15 samples was also annotated independently using the same SnpEff setup. These annotated VCFs were screened for the three established benzimidazole resistance-associated SNPs in the β-tubulin isotype 1 gene (HCON_00005260), which cause substitutions F167Y, E198A and F200Y [45, 54]. For detected SNPs, the resistance-associated allele frequency was calculated from FreeBayes read counts as AO / (RO + AO) × 100, where AO and RO represent the numbers of reads supporting the alternative and reference alleles, respectively [42].

For the three most divergent farm pairs, significantly differentiated SNPs were extracted from the three merged and annotated VCF files (bcftools view). One-to-one orthologues in *Caenorhabditis elegans* (annotation version WS290) were identified for all *H. contortus* genes harbouring these SNPs using the WormBase ParaSite BioMart web tool [55]. SNPs annotated as intergenic, upstream, downstream, or mapping to multiple genes were excluded, retaining only uniquely assigned, gene-based SNPs for downstream analysis. Gene Ontology (GO) enrichment analysis was performed using the g:Profiler g:GOSt web tool [56] with *C. elegans* WS290 as the reference organism. The statistical domain was restricted to annotated genes, and statistical significance was determined using the g:SCS multiple testing correction method (adjusted P < 0.05). For genes contributing to significantly enriched GO terms, both SNP impact categories (HIGH, MODERATE, LOW, MODIFIER) and predicted functional consequences, as annotated by SnpEff [52] based on Sequence Ontology terms [57], were further examined.

## Results

### Whole genome sequencing and mapping

Sequencing generated approximately 1.497 billion paired-end reads (220.6 Gb) across 17 samples. After trimming, 1.454 billion paired-end reads (97.1%) were retained, with a mean of 85.5 million paired-end reads per sample (range: 66.9–112.9 million). For most samples, the primary mapping rate averaged 76.5% (range: 52.7–89.1%). Across nuclear chromosomes, the mean breadth of coverage was 96.3% (range: 89.1–98.4%), and the mean depth was 64.3X (range: 32.8–92.0X), excluding the mitochondrial genome (Tables S1, S2). Two pools showed markedly poorer performance: sample M (primary mapping rate 10.0%, breadth of coverage 42.5%, mean depth 4.18X) and sample J (17.9%, 92.1%, 9.9X, respectively). These two samples were therefore excluded from downstream analyses.

### Genomic differentiation between farms and replicates

**Fig. 3:**
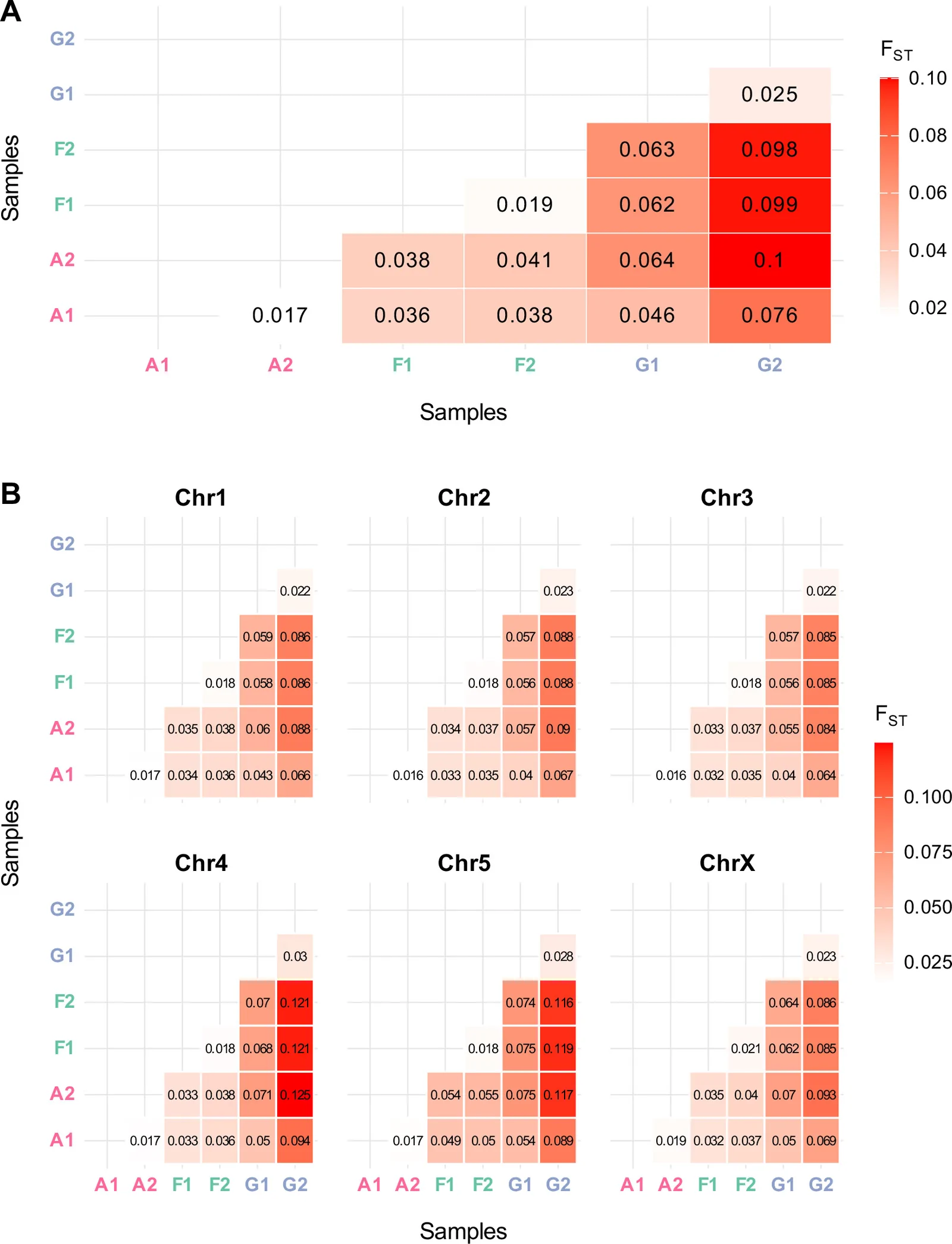
Within-farm genetic differentiation among biological *H. contortus* population replicates (sliding-window F_ST_, 10 kb window size with 5 kb step size). (**A**) Genome-wide pairwise F_ST_ between replicates from the farms A, F and G. (**B**) Chromosome-wide pairwise F_ST_ between replicates from the farms A, F and G (chromosomes 1-5 and X). Biological replicates are indicated as follows: samples A1 and A2 from Farm A (pink), samples F1 and F2 from Farm F (green), and samples G1 and G2 from Farm G (purple). Each cell reports an F_ST_ value; The opacity of the red colour cell fill indicates the degree of differentiation

**Fig. 4:**
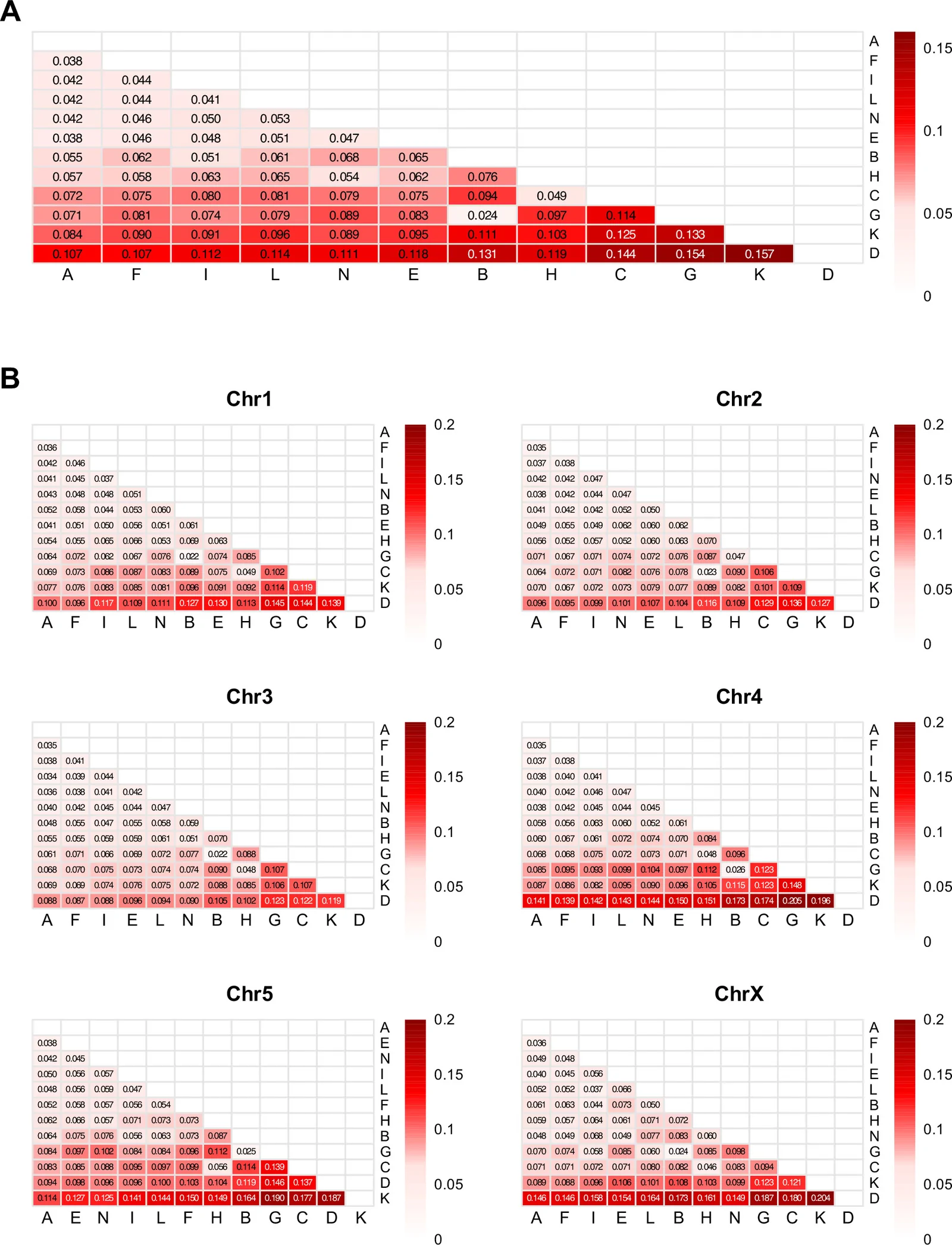
Between-farm genetic differentiation among *H. contortus* populations (sliding-window F_ST_, 10 kb window size with 5 kb step size). (**A**) Genome-wide pairwise F_ST_ between *H. contortus* populations from all 12 farms. (**B**) Chromosome-wide pairwise F_ST_ between *H. contortus* populations from all 12 farms (chromosomes 1-5 and X). For farms A, F, and G, average F_ST_ values of the two biological replicates were used; each cell reports the pairwise F_ST_ value, sorted in an increasing manner, with the opacity of the red colour cell fill indicating the degree of differentiation

**Fig. 5:**
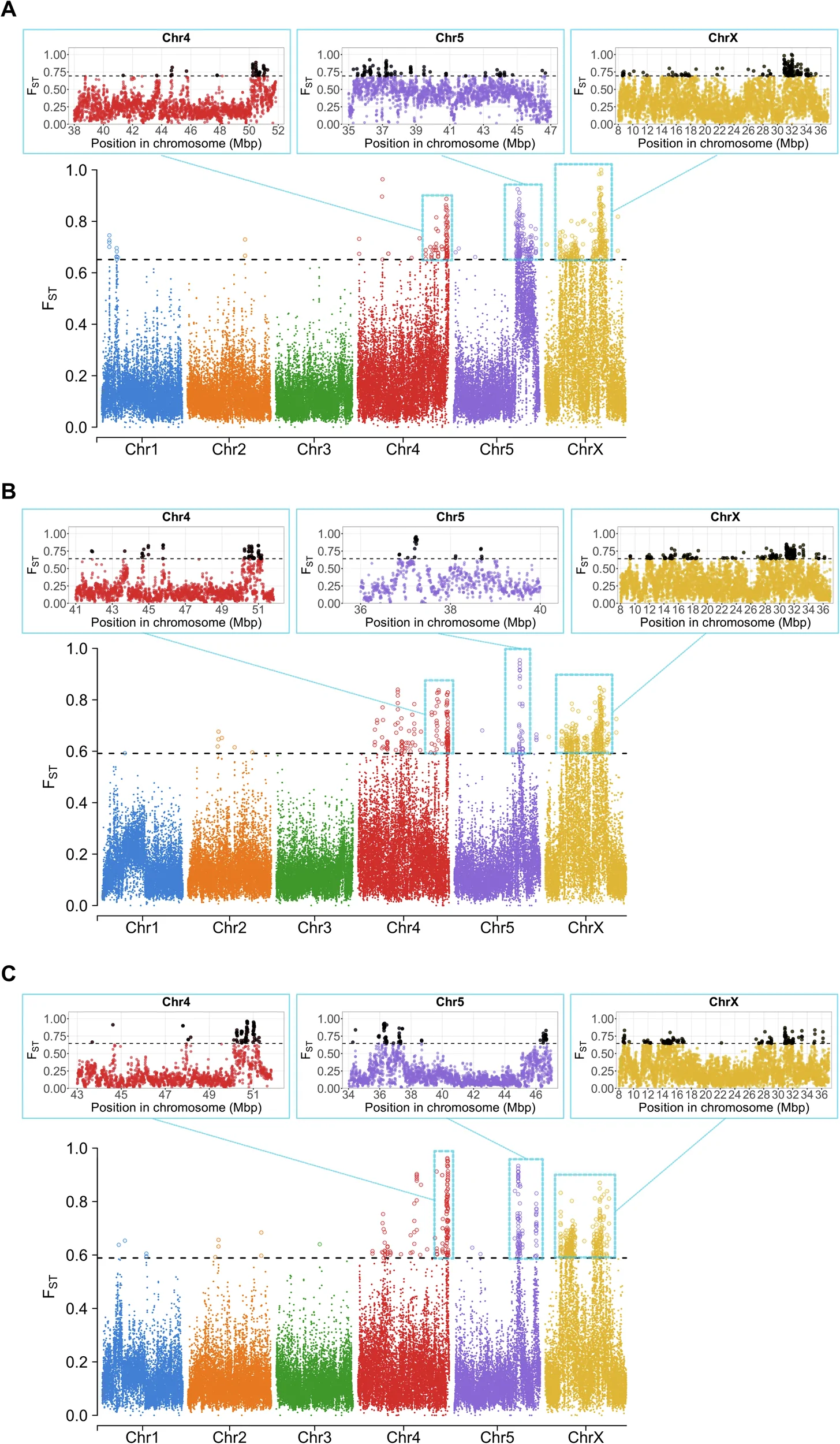
Sliding-window F_ST_ analysis for the three most divergent *H. contortus* farm population pairs: (**A**) farms D and K, (**B**) farms D and G, (**C**) farms D and C (10 kb window size with 5 kb step size). Each point represents an F_ST_ value for a genomic window, plotted at the midpoint of that window along six chromosomes. The horizontal dashed lines indicate the top 1% F_ST_ thresholds (A: 0.65, B: 0.59, C: 0.58), and windows exceeding these thresholds were considered highly differentiated. A total of 552 highly differentiated windows were identified for each population pair, and their distributions across chromosomes 1, 2, 3, 4, 5 and X, respectively, are shown in Table S4. The enlarged panels above each main plot show the main differentiated regions on chromosomes 4, 5, and X, with highly differentiated windows highlighted in black

We first compared genome-wide and chromosome-specific pairwise F_ST_ values based on a sliding-window approach, among all 12 *H. contortus* farm populations. The three tested window and step-size combinations yielded broadly consistent population patterns of differentiation (Figs. 3–5, S1–S6). The mean number of SNPs per window increased from 68 at 1 kb, to 590 at 10 kb, and 5756 at 100 kb window size, while the median F_ST_ IQR decreased from 0.052 to 0.041, and 0.028, respectively (Table S3). Based on these comparisons, we proceeded with a 10 kb window size and 5 kb step size. Within farms where biological replicate samples were available (farms A, F, and G), replicate pairs showed lower genomic divergence at both genome-wide and chromosome-specific levels than non-replicate pairs, as expected for replicated samples derived from the same population (Fig. 3; Figures S1, S2). With the 10 kb window size, genome-wide F_ST_ values ranged from 0.017 to 0.025 for replicate pairs, compared with 0.036 to 0.100 for non-replicate comparisons within farms A, F, and G. Corresponding chromosome-specific F_ST_ values ranged from 0.017 to 0.030 versus 0.032 to 0.125 (Fig. 3). Between-farm population comparisons consistently identified three most divergent pairs, farms D–K, D–G, and D–C (Fig. 4; Figures S3, S4), with genome-wide F_ST_ values of 0.144–0.157 and chromosome-specific F_ST_ values of 0.119–0.205 based on a 10 kb window size (Fig. 4). Furthermore, for these population pairs, chromosome-specific F_ST_ values were particularly higher on chromosomes 4, 5, and X (F_ST_ = 0.174–0.205) than those on the other chromosomes (F_ST_ = 0.119–0.145) (Fig. 4).

Subsequent analyses focused on identifying genomic regions underlying the pronounced differences observed in the three most divergent pairs. Using the top 1% of genome-wide sliding-window F_ST_ values as a threshold (farms D–K: F_ST_ > 0.65; farms D–G: F_ST_ > 0.59; farms D–C: F_ST_ > 0.58), we identified 552 highly differentiated 10 kb windows for each pairwise comparison. Most of these windows were located on chromosomes 4, 5 and X, with chromosome X consistently harbouring the largest proportion (farms D–K:53.1%; farms D–G: 65.6%; farms D–C: 58.3%), followed by chromosomes 5 (29.3%, 8.5%, 16.7%) and 4 (15.4%, 24.6%, 23%) (Fig. 5, Table S4).

Across the 15 retained samples, a total of 16,257,379 SNPs were identified for the per-SNP F_ST_ analysis, corresponding to approximately 589.8 SNPs per 10 kb sliding window. For the three most divergent farm population pairs (farms D–K, D–G, and D–C), FET was applied to the predefined highly differentiated SNPs and identified 25,304, 24,362, and 25,370 significantly differentiated SNPs, respectively (Bonferroni-adjusted P < 0.05), representing approximately 0.15% of all SNPs in each pair (Table 2). Consistent with the sliding-window F_ST_ analysis, these significantly differentiated SNPs were mainly concentrated on chromosomes 4, 5 and X, although their relative contributions varied among the three pairs (Table S5).

**Table 2:** Summary of SNPs and gene counts across pairwise farm population comparisons

Farm population pairs	No. of highly differentiated SNPs	No. of significantly differentiated SNPs	No. of significantly differentiated SNPs with annotation	No. of *H. contortus* genes with SNPs	No. of one-to-one *C. elegans* orthologs	No. of one-to-one *C. elegans* orthologs with HIGH/MODERATE SNPs	No. of one-to-one *C. elegans* orthologs with HIGH/MODERATE impact SNPs contributing to enriched GO terms
D vs K	26091	25304	15315	334	99	31	23
D vs G	24567	24362	15993	300	96	29	17
D vs C	26675	25370	16307	304	103	35	3

### Population structure

**Fig. 6:**
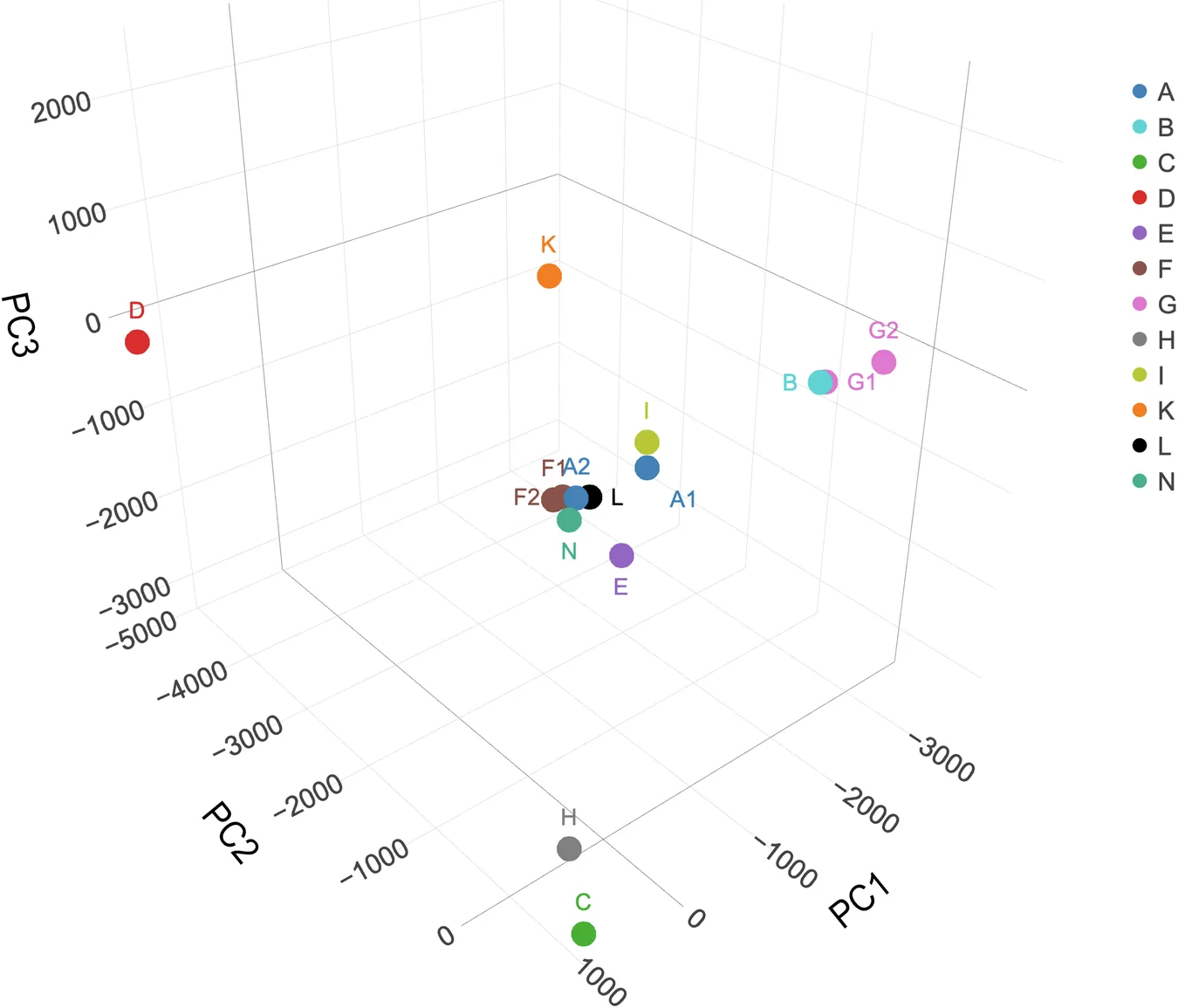
PCA of genome-wide SNP allele frequencies across the 15 *H. contortus* samples from 12 farms (Farms A, F, and G, each contributed two biological replicates). The first three principal components (PC) are visualised in the 3D scatter plot, with PC1, PC2, and PC3 explaining 16.4%, 13.3%, and 11.9% of the variance, respectively. An interactive 3D PCA plot is available at https://github.com/nxl365/hcontortus-pooled-wgs_2025.git

PCA was conducted on a subset of 14,444,526 genome-wide SNPs to reveal population structure among all 15 samples from 12 farms. The first principal component (PC1) explained 16.4% of the variance, while PC2 and PC3 explained 13.3% and 11.9% of the variance, respectively. Samples formed five major clusters, with most farms grouping together within the main cluster. Farm populations D, K, B–G, and C–H each formed separate clusters distinct from the main group, consistent with their higher levels of differentiation based on genome-wide F_ST_ patterns. Replicates from farms A, F, and G each clustered together, showing high within-farm consistency (Fig. 6; an interactive three-dimensional (3D) PCA plot is available on GitHub https://github.com/nxl365/hcontortus-pooled-wgs_2025.git).

### Genomic diversity within farms

**Fig. 7:**
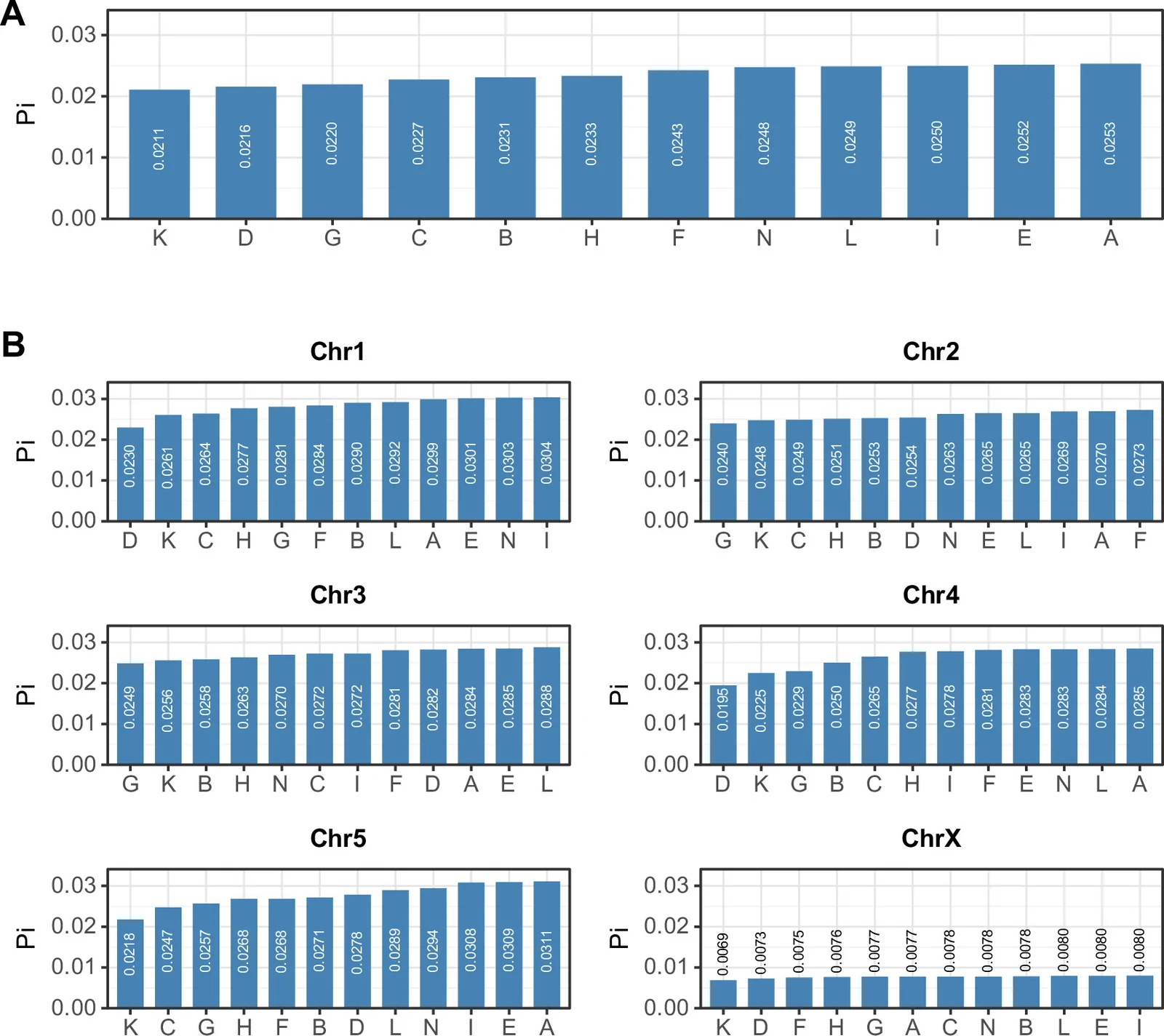
Nucleotide diversity (Pi) estimates for the 12 *H*. *contortus* farm populations; (**A**) genome-wide Pi estimates, (**B**) chromosome-wide Pi estimates (chromosomes 1–5 and X). Pi values are displayed numerically in each bar. For farms A, F, and G, average Pi values of the two replicates were used

Genome-wide Pi, summarised as weighted means across windows, was high and comparable across the 12 farm populations, ranging from 0.0211 on farm K to 0.0253 on farm A (Fig. 7). The lowest genome-wide Pi values were observed in farm populations K, D, G, and C (0.0211–0.0227), whereas farm populations A, E, I and L showed the highest diversity (0.0250–0.0253). At the chromosome level, although the exact ranking differed among the chromosome-wide Pi values, farm populations with the highest or lowest nucleotide diversity were often among the same few populations identified above; chromosome X exhibited markedly lower nucleotide diversity (0.0069–0.0080) than autosomes (0.0195–0.0311) in all farm populations (Fig. 7).

**Fig. 8:**
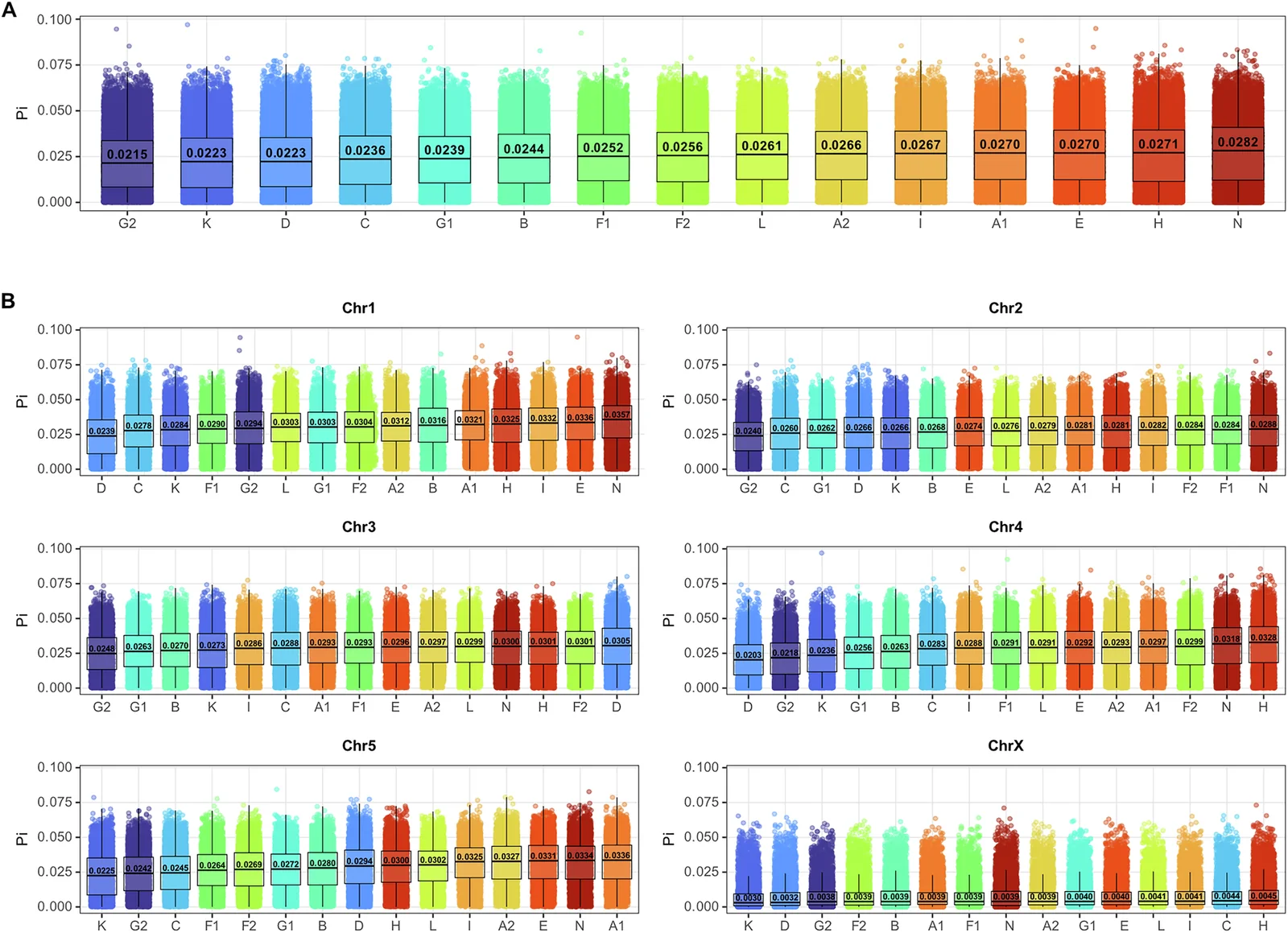
The distribution of window-based nucleotide diversity (Pi) estimates for the 15 *H*. *contortus* samples from 12 farms (Farms A, F, and G, each contributed two biological replicates); (**A**) Genome-wide distribution of Pi, (**B**) chromosome-wide distribution (chromosomes 1–5 and X) of Pi. Each dot represents a Pi value calculated for a 10 kb genomic window. The boxes represent the interquartile range (IQR), and the horizontal line within each box indicates the median window-based Pi value, which is also displayed numerically. Boxes are arranged in ascending order of median Pi from left to right, and a consistent colour was used for each sample across all plots

This overall pattern was also broadly reflected in the distributions of genome-wide and chromosome-wide window-based Pi values, although the correspondence was not exact for all farms. Biological replicates from farm populations A, F, and G consistently showed broadly similar distributions, as expected. Genome-wide median window-based Pi values ranged from 0.0215 to 0.0282 across samples, corresponding to a maximum difference of 0.0067, while the IQRs overlapped substantially (Fig. 8). Chromosome-wide Pi landscapes further showed that Pi varied across genomic windows within each sample, indicating an uneven distribution of nucleotide diversity along the genome. Across samples, autosomal Pi profiles were broadly similar, whereas chromosome X showed markedly lower diversity (Figure S7).

However, pairwise Wilcoxon Rank Sum tests detected statistically significant differences in the genome-wide window-based Pi distributions for most population comparisons (adjusted P-value < 0.05). Comparisons between farm populations with more similar distributions of genome-wide window-based Pi values (e.g., farms B and F) produced higher P-values than comparisons between populations with more distinct distributions (e.g., farms K and N) (Figure S8).

### Functional annotation and gene ontology

For the three most divergent farm population pairs, the numbers of significantly differentiated SNPs with functional annotations were 15,315, 15,993, and 16,307, respectively, whereas on average 9,140 SNPs per pair (~36%) lacked annotation (Table 2). Across all three pairs, the impact categories and predicted functional consequences of the annotated SNPs are shown in Tables S6 and S7. They were predominantly classified as LOW/MODIFIER impact variants (approximately 97%), with only 3% classified as HIGH/MODERATE impact variants. The lower proportion of HIGH/MODERATE impact variants is expected due to constrained natural selection, although the few ones found in this study could still potentially be important as they are represented by missense variants as well as modulating transcription start and stop sites. Uniquely assigned gene-based SNPs mapped to approximately 300 distinct *H. contortus* genes per comparison, corresponding to roughly 100 one-to-one *C. elegans* orthologues (Table 2).

GO enrichment results for the three farm population pairs are summarised in Tables 3–5. For the D-K comparison, twelve GO terms were significantly overrepresented among 99 genes, with 31 genes harbouring HIGH/MODERATE impact SNPs contributing to these enrichments. The D-G comparison yielded two significantly overrepresented GO terms among 96 genes, with 17 genes containing HIGH/MODERATE impact SNPs. In the D-C comparison, six GO terms were significantly enriched among 103 genes, with three genes harbouring HIGH/MODERATE impact SNPs. SNP counts and functional consequence classifications for genes harbouring HIGH/MODERATE impact SNPs are provided in Tables S8–S10.

**Table 3:** Enriched Gene Ontology terms for 99 orthologous *C. elegance* genes comprising significantly differentiated *H. contortous* SNPs in the comparison between farm populations D and K

GO term^a^	Term name	Term identity	Adjusted p-value	Term size^b^	Query size^c^	Intersection size^d^	Effective domain size^e^	Intersections^f^	Gene with HIGH/MODERATE impact SNPs^g^
GO:MF	Molecular function regulator activity	GO:0098772	0.0029	499	82	14	11705	*RRC-1, GCY-35, VAV-1, SYD-2, AAKG-4, C08G9.2, MTM-5, SIPA-1, AGS-3, F35B12.10, SOS-1, F43B10.1, EAT-17, RGA-8*	*C08G9.2, MTM-5, SIPA-1, SYD-2*
GO:MF	GTPase regulator activity	GO:0030695	0.0030	148	82	8	11705	*RRC-1, VAV-1, MTM-5, SIPA-1, AGS-3, SOS-1, EAT-17, RGA-8*	*MTM-5, SIPA-1*
GO:MF	Nucleoside-triphosphatase regulator activity	GO:0060589	0.0030	148	82	8	11705	*RRC-1, VAV-1, MTM-5, SIPA-1, AGS-3, SOS-1, EAT-17, RGA-8*	*MTM-5, SIPA-1*
GO:MF	Cation binding	GO:0043169	0.0154	1989	82	29	11705	*B0563.7, ZK563.7, CTBP-1, POD-2, GCY-35, ZTF-16, VAV-1, T04G9.4, AAKG-4, SGK-1, HECD-1, MTM-5, PKC-2, ODD-2, SGCA-1, SAD-1, Y92H12BL.1, DISL-2, NFU-1, PAR-4, Y1A5A.1, ZK287.1, RBX-1, AIP-1, F43B10.1, ADT-2, MEK-1, NHR-32, UNK-1*	*ADT-2, DISL-2, HECD-1, MTM-5, PAR-4, SGCA-1, ZK563.7*
GO:MF	Enzyme regulator activity	GO:0030234	0.0179	371	82	11	11705	*RRC-1, VAV-1, AAKG-4, C08G9.2, MTM-5, SIPA-1, AGS-3, SOS-1, F43B10.1, EAT-17, RGA-8*	*C08G9.2, MTM-5, SIPA-1*
GO:MF	Metal ion binding	GO:0046872	0.0337	1965	82	28	11705	*B0563.7, ZK563.7, CTBP-1, POD-2, GCY-35, ZTF-16, VAV-1, T04G9.4, SGK-1, HECD-1, MTM-5, PKC-2, ODD-2, SGCA-1, SAD-1, Y92H12BL.1, DISL-2, NFU-1, PAR-4, Y1A5A.1, ZK287.1, RBX-1, AIP-1, F43B10.1, ADT-2, MEK-1, NHR-32, UNK-1*	*ADT-2, DISL-2, HECD-1, MTM-5, PAR-4, SGCA-1, ZK563.7*
GO:BP	Multicellular organismal process	GO:0032501	0.0016	2343	74	34	11036	*POD-2, FKH-9, GCY-35, MEC-18, ZTF-16, VAV-1, UGGT-1, SYG-1, SYD-2, SAX-3, SGK-1, CEH-40, HECD-1, PTR-14, CAT-1, HOT-1, AGS-3, SAD-1, EOR-2, IMPT-1, LGC-22, STA-1, PAR-4, NDC-80, EGL-2, DPY-11, UBH-1, RBX-1, SOS-1, ACR-10, ADT-2, EAT-17, MEK-1, SEL-7*	*ADT-2, DPY-11, HECD-1, HOT-1, IMPT-1, LGC-22, MEC-18, NDC-80, PAR-4, PTR-14, SYD-2, SYG-1*
GO:BP	Signaling	GO:0023052	0.0095	2064	74	30	11036	*RRC-1, GCY-35, VAV-1, UGGT-1, SYG-1, SYD-2, SAX-3, SGK-1, HECD-1, SIPA-1, NPR-3, C24B5.1, PKC-2, GRK-1, SAD-1, EOR-2, LGC-22, STA-1, PAR-4, F35B12.10, UBH-1, RBX-1, SOS-1, ACR-10, FSHR-1, EAT-17, MEK-1, SEL-7, NPR-11, RGA-8*	*C24B5.1, FSHR-1, HECD-1, LGC-22, PAR-4, SIPA-1, SYD-2, SYG-1*
GO:BP	Cell communication	GO:0007154	0.0104	2073	74	30	11036	*RRC-1, GCY-35, VAV-1, UGGT-1, SYG-1, SYD-2, SAX-3, SGK-1, HECD-1, SIPA-1, NPR-3, C24B5.1, PKC-2, GRK-1, SAD-1, EOR-2, LGC-22, STA-1, PAR-4, F35B12.10, UBH-1, RBX-1, SOS-1, ACR-10, FSHR-1, EAT-17, MEK-1, SEL-7, NPR-11, RGA-8*	*C24B5.1, FSHR-1, HECD-1, LGC-22, PAR-4, SIPA-1, SYD-2, SYG-1*
GO:BP	Multicellular organism development	GO:0007275	0.0190	1379	74	23	11036	*POD-2, FKH-9, ZTF-16, VAV-1, UGGT-1, SYG-1, SYD-2, SAX-3, SGK-1, CEH-40, HECD-1, SAD-1, EOR-2, IMPT-1, STA-1, PAR-4, NDC-80, DPY-11, UBH-1, RBX-1, SOS-1, MEK-1, SEL-7*	*DPY-11, HECD-1, IMPT-1, NDC-80, PAR-4, SYD-2, SYG-1*
GO:BP	Biological regulation	GO:0065007	0.0302	44	74	47	11036	*RRC-1, CTBP-1, FKH-9, GCY-35, ZTF-16, VAV-1, UGGT-1, SYG-1, SYD-2, SAX-3, SGK-1, EBP-1, RBC-1, CEH-40, HECD-1, SIPA-1, NPR-3, C24B5.1, PKC-2, ODD-2, GRK-1, AGS-3, SAD-1, EOR-2, DISL-2, IMPT-1, LGC-22, STA-1, PAR-4, F35B12.10, NDC-80, EGL-2, DPY-11, UBH-1, RBX-1, SOS-1, ACR-10, FSHR-1, ADT-2, EAT-17, MEK-1, NHR-32, SEL-7, NPR-11, RGA-8, LGC-47, TWK-28*	*ADT-2, C24B5.1, DISL-2, DPY-11, EBP-1, FSHR-1, HECD-1, IMPT-1, LGC-22, NDC-80, PAR-4, RBC-1, SIPA-1, SYD-2, SYG-1*
GO:CC	Neuron projection	GO:0043005	0.0275	498	89	12	12880	*GCY-35, MEC-18, SYG-1, SYD-2, CAT-1, PKC-2, HOT-1, SAD-1, LGC-43, LGC-22, ACR-10, LGC-47*	*HOT-1, LGC-22, LGC-43, MEC-18, SYD-2, SYG-1*

^a^Main category of gene ontology terms; Molecular Function (GO:MF), Biological Process (GO:BP), and Cellular Component (GO:CC)

^b^Number of background genes annotated to this term

^c^Number of genes in our input list used for enrichment

^d^Count of our input genes that are annotated to this term

^e^Size of the background “statistical domain” used for testing

^f^The actual genes that are annotated to this term from our input (one-to-one *C.elegans* orthologs)

^g^Subset of the actual genes that carry MODERATE/HIGH impact SNPs from our input

**Table 4:** Enriched Gene Ontology terms for 96 orthologous *C. elegance* genes comprising significantly differentiated *H. contortous* SNPs in the comparison between farm populations D and G

GO term^a^	Term name	Term identity	Adjusted p-value	Term size^b^	Query size^c^	Intersection size^d^	Effective domain size^e^	Intersections^f^	Gene with HIGH/MODERATE impact SNPs^g^
GO:BP	nervous system development	GO:0007399	0.0199	434	72	12	10936	*FKH-9, MIG-2, SYG-1, SYD-2, CEH-40, DPFF-1, UNC-3, SAD-1, EOR-2, IMPT-1, MEK-1, UNC-6*	*IMPT-1, SYD-2, SYG-1*
GO:CC	intracellular anatomical structure	GO:0005622	0.0149	6933	85	63	12900	*B0563.7, NSTP-10, FKH-9, GEI-15, BPL-1, MDT-6, EPG-7, GCY-35, QUI-1, MIG-2, T04G9.4, UGGT-1, Y57E12AL.6, SYD-2, DYC-1, SGK-1, RBC-1, NSTP-2, ASNS-2, CEH-40, HECD-1, MIGA-1, MTM-5, F26A10.2, TWNK-1, SIPA-1, PTR-14, HDA-11, EFL-2, DRH-1, DPFF-1, PKC-2, C25H3.15, ODD-2, T25B9.1, SGCA-1, UNC-3, GRK-1, AJM-1, F13E6.2, EOR-2, DHS-29, BED-3, Y57G11C.33, C24D10.6, UNC-82, DPF-5, GTA-1, COX-18, DISL-2, IMPT-1, MASK-1, NFU-1, MCM-2, ZK287.1, MEK-1, NHR-32, MBK-1, RRF-3, SEL-7, UNC-6, UNK-1, HOSL-1*	*DISL-2, DRH-1, GEI-15, HECD-1, IMPT-1, MASK-1, MCM-2, MTM-5, NHR-32, PTR-14, QUI-1, RBC-1, RRF-3, SGCA-1, SYD-2, UNC-82*

^a^Main category of gene ontology terms; Biological Process (GO:BP), and Cellular Component (GO:CC)

^b^Number of background genes annotated to this term

^c^Number of genes in our input list used for enrichment

^d^Count of our input genes that are annotated to this term

^e^Size of the background “statistical domain” used for testing

^f^The actual genes that are annotated to this term from our input (one-to-one *C.elegans* orthologs)

^g^Subset of the actual genes that carry MODERATE/HIGH impact SNPs from our input

**Table 5:** Enriched Gene Ontology terms for 103 orthologous *C. elegance* genes comprising significantly differentiated *H. contortous* SNPs in the comparison between farm populations D and C

GO term^a^	Term name	Term identity	Adjusted p-value	Term size^b^	Query size^c^	Intersection size^d^	Effective domain size^e^	Intersections^f^	Gene with HIGH/MODERATE impact SNPs^g^
GO:MF	GTPase regulator activity	GO:0030695	0.0003	148	80	9	11705	*RRC-1, VAV-1, RPI-2, MTM-5, TBC-19, AGS-3, F53A10.2, EAT-17, RGA-8*	*RPI-2*
GO:MF	nucleoside-triphosphatase regulator activity	GO:0060589	0.0003	148	80	9	11705	*RRC-1, VAV-1, RPI-2, MTM-5, TBC-19, AGS-3, F53A10.2, EAT-17, RGA-8*	*RPI-2*
GO:MF	GTPase activator activity	GO:0005096	0.0057	79	80	6	11705	*RRC-1, RPI-2, TBC-19, F53A10.2, EAT-17, RGA-8*	*RPI-2*
GO:MF	molecular function regulator activity	GO:0098772	0.0109	499	80	13	11705	*RRC-1, GCY-35, VAV-1, SYD-2, DYC-1, RPI-2, C08G9.2, MTM-5, TBC-19, AGS-3, F53A10.2, EAT-17, RGA-8*	*C08G9.2, RPI-2, SYD-2*
GO:MF	GTPase activity	GO:0003924	0.0125	299	80	10	11705	*RRC-1, MIG-2, VAV-1, RPI-2, MTM-5, TBC-19, AGS-3, F53A10.2, EAT-17, RGA-8*	*RPI-2*
GO:MF	enzyme regulator activity	GO:0030234	0.0149	371	80	11	11705	*RRC-1, VAV-1, DYC-1, RPI-2, C08G9.2, MTM-5, TBC-19, AGS-3, F53A10.2, EAT-17, RGA-8*	*C08G9.2, RPI-2*

^a^Main category of gene ontology term; Molecular Function (GO:MF)

^b^Number of background genes annotated to this term

^c^Number of genes in our input list used for enrichment

^d^Count of our input genes that are annotated to this term

^e^Size of the background “statistical domain” used for testing

^f^The actual genes that are annotated to this term from our input (one-to-one *C.elegans* orthologs)

^g^Subset of the actual genes that carry MODERATE/HIGH impact SNPs from our input

Separately, we screened the three established benzimidazole resistance-associated SNPs. Those causing amino acid substitutions F167Y and E198A were not detected in any sample, whereas F200Y was detected in 12 out of the 15 samples, with allele frequencies ranging from 43.2% to 100.0% (Table S11).

## Discussion

This study presents the genome-wide characterisation of the genetic variation within and between *H. contortus* populations isolated from naturally infected sheep on Swedish farms. Using Pool-seq, we found that the multiple field-derived isolates exhibit relatively high nucleotide diversity within populations, but comparably low genomic differentiation between most population pairs. These findings are consistent with the large effective population size and high fecundity of *H. contortus*, together with substantial gene flow. However, three population pairs exhibited higher genomic differentiation and lower nucleotide diversity, which may reflect additional farm-specific factors. In addition, genetic differentiation was not evenly distributed across the genome but concentrated on chromosomes 4, 5, and X. Furthermore, significantly differentiated SNPs were associated with a limited set of annotated genes, enriched for regulatory, signalling and nervous system-related functions, providing candidate functional patterns for future investigation. Together, these findings provide a genome-wide population genomic baseline for Swedish *H. contortus*. They also provide a useful resource for future surveillance of anthelmintic resistance and for comparisons with populations from other geographical regions.

### Utility and limitations of Pool-seq

Pool-seq provided a practical and cost-effective method for describing broad population structure and genomic differentiation in this study. With a sufficiently large pool size as well as adequate sequencing depth and coverage, Pool-seq can provide reliable estimates of population allele frequencies [36]. However, the Pool-seq approach also has some limitations, such as unequal DNA contribution among individuals that may arise from technical errors during DNA quantification or pooling. Reduced precision for low-frequency variants and rare alleles is another limitation, as they can be difficult to distinguish from sequencing errors [36, 58]. Because F_ST_, Pi, and PCA in this study all relied on allele frequency information, these sources of uncertainty may affect their estimates, and may therefore influence the identification of differentiated SNPs and windows. These signals should be interpreted as candidate signals of genomic differentiation rather than direct evidence of local adaptation or selection. Short-read Pool-seq also presents limited capacity to infer individual heterozygosity, long-range individual haplotypes, and linkage disequilibrium. Future studies using individual whole genome sequencing and/or long-read Pool-seq could help overcome these limitations.

### Sample exclusion and species misclassification

Two samples (from farms J and M) were excluded because of low mapping rates (Tables S1, S2), indicating that a large proportion of reads failed to align with the *H. contortus* reference genome. However, the DNA and sequencing read quality of these two samples were comparable to those of the other samples (data not shown), suggesting that poor quality was unlikely to explain the low mapping rates. One plausible explanation is that these samples contained substantial amounts of DNA from species other than *H. contortus*. Although *H. contortus* was identified by microscopy during sample selection, discrepancies may occur between molecular and microscopy-based species identification [59, 60]. However, the species composition will be investigated in future studies.

### Window-size effects and replicate consistency

We investigated genomic variation within and between Swedish *H. contortus* populations using PCA and the population genetic parameters F_ST_ and Pi. To assess the impact of different window sizes on detecting patterns of differentiation between populations, we compared 1, 10 and 100 kb F_ST_ windows. Genome-wide and chromosome-wide differentiation patterns were relatively consistent across the three window sizes (Figs. 3–5, S1–S6), indicating that the regions showing the highest differentiation signals were consistently detected and localised despite differences in the chosen window size. However, window size affected the local resolution and variability of the F_ST_ profiles. The 1 kb window size provided a finer genomic resolution but contained fewer SNPs per window (68) and showed greater F_ST_ variability (Median F_ST_ IQR = 0.052), whereas the 100 kb window size contained substantially more SNPs per window (5,756) and produced smoother F_ST_ profiles (Median F_ST_ IQR = 0.028), but at the cost of lower local genomic resolution (Table S3; Figures S5, S6). We therefore adopted a 10 kb window size for downstream analyses, providing a practical balance between genomic resolution and stochastic noise (Table S3; Fig. 5). The same window size has also been used in previous *H. contortus* studies [10, 12, 61]. Subsequently, we applied the same 10 kb window size to the within-population comparisons (Pi).

After evaluating the effects of window size, we examined whether the variation estimates between biological replicates for farm populations (A, F and G) were properly reflected. Unsurprisingly, replicates from the same farm population clustered together in the PCA (Fig. 6), had similar Pi distributions (Fig. 8), and exhibited overall lower F_ST_ values than non-replicate sample comparisons (Fig. 3). As these biological replicates were derived from different sheep from the same farm and processed independently, their high similarity provides an internal assessment of the reproducibility and robustness of our sampling.

### Genome-wide patterns of population differentiation and diversity

The relatively low genome-wide differentiation among most farm populations (F_ST_ < 0.1) [62] (Fig. 4) indicates a largely similar population structure shared among the *H. contortus* populations. This notion is further reinforced by the PCA analysis, which showed most samples clustering tightly together (Fig. 6). In contrast, nucleotide diversity estimates indicate substantial genomic diversity within the studied *H. contortus* populations (genome-wide Pi values ranged from 0.0211 to 0.0253; autosomal chromosome-wide Pi values ranged from 0.0195 to 0.0311) (Fig. 7). This is broadly consistent with previous reports of high nucleotide diversity in *H. contortus* [61, 63]. Doyle et al. [61] reported mean autosomal Pi values of ~0.022–0.038 across the five autosomes in three laboratory-maintained isolates, while Khan et al. [63] reported genome-wide average Pi values of ~0.029–0.033.

Although the distributions of window-based Pi were broadly similar across farm populations, there were relatively small differences in medians and substantial overlap in IQRs (Fig. 8), pairwise Wilcoxon Rank Sum tests still identified significant differences for most population comparisons (adjusted P-value < 0.05) (Figure S8). This, however, is more likely due to the large number of genomic windows used in the analysis, which can make even small shifts statistically significant without necessarily being biologically meaningful. Therefore, the magnitude and overlap of the Pi distributions were considered more informative than statistical significance alone. Genome-wide nucleotide diversity was generally high across farms, while comparisons of window-based Pi distributions suggested that differences among farms were overall subtle.

This overall pattern is consistent with other national-scale studies, which report similarly low between-population differentiation and high within-population diversity in *H. contortus* from China [17, 64], Bangladesh [8, 15] and Thailand [28]. The high genetic diversity of *H. contortus* is thought to arise from its large population size and high fecundity [12]. In Sweden, rams exchanged between farms have been shown to carry *H. contortus*, potentially facilitating gene flow among parasite populations [65]. At local and regional scales, genetic differentiation among populations is often relatively low, consistent with high gene flow within countries or continents [14, 30], where host movement together with their parasites is common. By contrast, global surveys have revealed clearer divergence and clustering among populations from different continents, indicating that population structure can become more pronounced at broader geographical scales, where livestock movement and trade are more limited [4, 8, 12, 13, 30].

### Populations with higher F_ST_ and lower Pi

We identified three farm population pairs, D–K, D–G and D–C, which consistently showed higher F_ST_ values than others (Fig. 4; Figures S3, S4). Furthermore, our PCA based on genome-wide SNP allele frequencies showed that these farm populations were markedly separated from the main cluster (Fig. 6). Interestingly, geographic distance alone does not seem to explain this variation. Based on visual inspection, the three most divergent farm population pairs were not the most geographically distant and, in some cases, were closer to each other than pairs showing lower differentiation (Fig. 1). However, formal isolation-by-distance testing was not performed. Instead, the observed differences could potentially be influenced by the history of the hosts, leading to the inadvertent import of parasites and admixture with the local *H. contortus* population [12]. In that case, farm-to-farm genomic structure within Sweden may be shaped not only by local farm factors, but also by anthropogenic animal movements between farms [66], shared treatment histories and animal/parasite introduction routes. This in turn could explain why some farms (e.g. farm D) remain more differentiated than others.

In addition to being the most highly differentiated pairs, farm populations K, D, G and C also displayed the lowest genome-wide within-population diversity estimates (Figs. 4, 7), indicating that these populations may have undergone local drift or experienced a population bottleneck. Alternatively, they may have been influenced by selective processes, including farm-specific management and treatment histories [12, 23]. However, farm metadata, including information on treatment history, animal movement and introduction, and grazing management, were not systematically available for the sampled farms. As the present study was not designed to disentangle demographic and selective effects, these explanations remain tentative and should be explored further in future work integrating genomic data with farm metadata.

### Chromosome-specific patterns of population differentiation and diversity

In our study, genetic differentiation estimates were not evenly distributed across the genome. For the three most differentiated farm population pairs, chromosome‑specific F_ST_ values were higher on chromosomes 4, 5, and X (Fig. 4). The most highly differentiated genomic windows (Fig. 5; Table S4) and significantly differentiated SNPs cluster on these chromosomes (Table S5). Notably, in all three population pairs, a subset of the highly differentiated windows overlapped previously reported regions on chromosomes 4 and 5. On chromosome 5, several windows fell within the ~37–42 Mb region previously identified as containing a major ivermectin-resistance quantitative trait locus (QTL) [61]. Baltrušis et al. [10] also reported a major F_ST_ peak beginning at ~37.4 Mb in a Swedish field population in a pre- versus post-ivermectin treatment comparison. On chromosome 4, some highly differentiated windows overlapped the terminal ~40–51 Mb region where Baltrušis et al. [10] reported outlier signals in the same study. The correspondence with previously reported regions suggests that historical treatment-mediated selection could have contributed to the observed differentiation. However, the chromosome 4 signal remains unresolved [10], and without treatment histories or resistance phenotypes, positional overlap alone cannot demonstrate that the same loci or selective processes are involved. This caution is also supported by Doyle et al. [61], who argued that substantial differentiation among *H. contortus* populations could reflect population structure arising from independent evolutionary histories and limited interbreeding, rather than drug-related selection. Furthermore, Doyle et al. [61] showed that limited recombination around a QTL at chromosome 5 resulted in large divergent haplotype blocks. To what extent recombination contributed to the chromosome-specific patterns observed here remains unresolved.

Chromosome X showed elevated F_ST_ in the three most divergent population pairs (Figs. 4, 5) and lower Pi than the autosomes across all populations (Figs. 7, 8, S7). Previous studies have similarly identified regions of elevated F_ST_ on chromosome X and reduced nucleotide diversity relative to the autosomes [10, 16, 63]. This pattern may partly reflect sex-chromosome-specific evolutionary dynamics, including sex-biased reproductive success and resulting differences in effective population size between males and females. Additionally, reduced recombination on chromosome X is expected, as recombination occurs only during female gametogenesis. Interestingly, the region of chromosome X showing relatively higher F_ST_ and lower Pi in our data (Fig. 5, S7) broadly overlaps the previously described ~8–35 Mb repeat-enriched region [16]. However, additional caution is required when interpreting the results. *H. contortus* females are XX whereas males are XO, and the sex ratio of larvae in pools was not controlled, which could affect the representation of chromosome X. Nevertheless, the sequencing depth on chromosome X was comparable to that on autosomes in our study, indicating no clear systematic under-representation (Table S2). Technical artefacts cannot be excluded, as repetitive regions may be difficult to resolve fully in a linear reference genome [16] and to map reliably using short reads; indeed, chromosome X showed somewhat lower mapping quality than autosomes in our data (Table S2). Overall, the observed pattern may reflect a combination of demographic history, chromosomal evolutionary processes, regional genomic features, and technical artefacts, and should therefore be interpreted with caution.

### Functional enrichment of genes with differentiated SNPs

To explore the potential functions of genes containing significantly differentiated SNPs, we performed GO enrichment analyses. Functional inference from *C. elegans* orthology may be informative in *H. contortus*, given the high level of conservation of orthologous genes between the two species, despite extensive rearrangement of gene order [16]. Across the three most divergent population pairs, several GO terms were repeatedly enriched: regulatory and signalling-related terms, such as GTPase regulator activity, enzyme regulator activity, signalling and cell communication, as well as nervous-system-related terms.

In *H. contortus*, previous studies have shown that conserved dauer-associated signalling pathways directly contribute to the larval developmental transitions [67, 68]. Similar functional categories have also been reported with drug-response and neurotransmission-related functions mainly associated with drug-influenced selection signals, and dauer-pathway components linked to climate-related differentiation [12]. Studies in other nematodes provide further biological context. In *C. elegans*, GTPase regulatory pathways are involved in presynaptic development through Rap/Rac signalling [69], while Rab GTPases contribute to membrane trafficking and developmental processes [70]. In *Strongyloides stercoralis*, sensory and neural functions are relevant to thermosensory host seeking [71].

However, our results should be interpreted with caution, as most annotated variants belonged to the LOW/MODIFIER category, many SNPs remained unannotated, several enriched GO terms were very broad, and some may be driven by only a small number of genes. In addition, functional inference partly relies on *C. elegans* orthology, while *H. contortus*-specific functional annotation remains incomplete, and differentiated SNPs do not necessarily represent causal variants. Nevertheless, these findings suggest that the highest differentiation in the three population pairs of *H. contortus* tends to be associated with genes involved in regulatory, signalling, and nervous-system-related functions, but they should be regarded as exploratory and hypothesis-generating rather than definitive explanations of population divergence. Distinguishing whether these patterns reflect selection, drift, or farm-specific effects will require future analyses with phenotype-linked data such as drug-response phenotypes, treatment history, and farm-management metadata.

As an additional characterisation of genetic variation among populations, the known benzimidazole resistance-associated allele (amino acid substitution F200Y) was detected at varying frequencies across most samples (Table S11). This is in line with a previous report showing that F200Y is widespread in Swedish *H. contortus* populations [54]. However, these data were not further interpreted in relation to population differentiation because the phenotypic resistance data and treatment histories were unavailable in our study.

## Conclusion

Overall, this work provides the first genome-wide baseline for field-derived *H. contortus* populations across multiple Swedish sheep farms. It shows that Pool-seq is a useful method for describing broad population genomic patterns in this species. The Swedish populations appeared broadly genetically connected, although some farm population pairs showed greater divergence, consistent with substantial gene flow alongside plausible farm-specific effects. As *H. contortus* populations are highly diverse, differentiated genomic regions should be interpreted with caution. This is particularly important for future anthelmintic resistance research, where genomic scans for candidate resistance variants may be influenced by the broader population-genomic background and drug-induced selection, and therefore our present study offers a valuable baseline for interpretation. These findings are also relevant for parasite surveillance and control, as connectivity among farms may facilitate the spread of adaptive variants, including resistance-associated alleles. This study lays an important foundation for future research on the genetic basis of adaptation and drug resistance in *H. contortus*.

## Supplementary information

Below is the link to the electronic supplementary material.

Additional file 1: Supplementary figures.

Additional file 2: Supplementary tables.

## Data Availability

The SNP datasets generated and analysed during the current study are available in the European Nucleotide Archive under the study accession number PRJEB112099. The H. contortus reference genome and GFF3 annotation file (WormBase release 2021-04) used during the current study are available in the WormBase repository (https://parasite.wormbase.org/Haemonchus_contortus_prjeb506/Info/Index/). Code supporting this study is available on GitHub (https://github.com/nxl365/hcontortus-pooled-wgs_2025.git).

## Abbreviations

BAM: Binary Alignment/Map format
BCF: Binary Call Format
BWA-MEM: Burrows Wheeler Aligner - Maximal Exact Matches
EPG: Eggs per gram of faeces
FASTQ: Raw sequencing reads file format
FET: Fisher’s exact test
F_ST_: Fixation index
GFF3: General Feature Format version 3
GO: Gene Ontology
IQR: Interquartile range
mtDNA: Mitochondrial DNA
PC: Principal component
PCA: Principal component analysis
Pi: Nucleotide diversity
Pool-seq: Pooled-sample whole genome sequencing
QTL: Quantitative trait locus
SAM: Sequence Alignment/Map format
SNP: Single nucleotide polymorphism
VCF: Variant Call Format
WGS: Whole genome sequencing
