# Supplementary material for: Whole genome pool sequencing reveals genomic variation in field Haemonchus contortus isolates in Sweden
Source: BMC Genomics. 2026 Oct 17;27:870. doi: 10.1186/s12864-026-13449-5 (PMC13678931; doi:10.1186/s12864-026-13449-5)
Supplement: Supplementary file 1 [file 12864_2026_13449_MOESM1_ESM.pdf]

Supplementary Figures

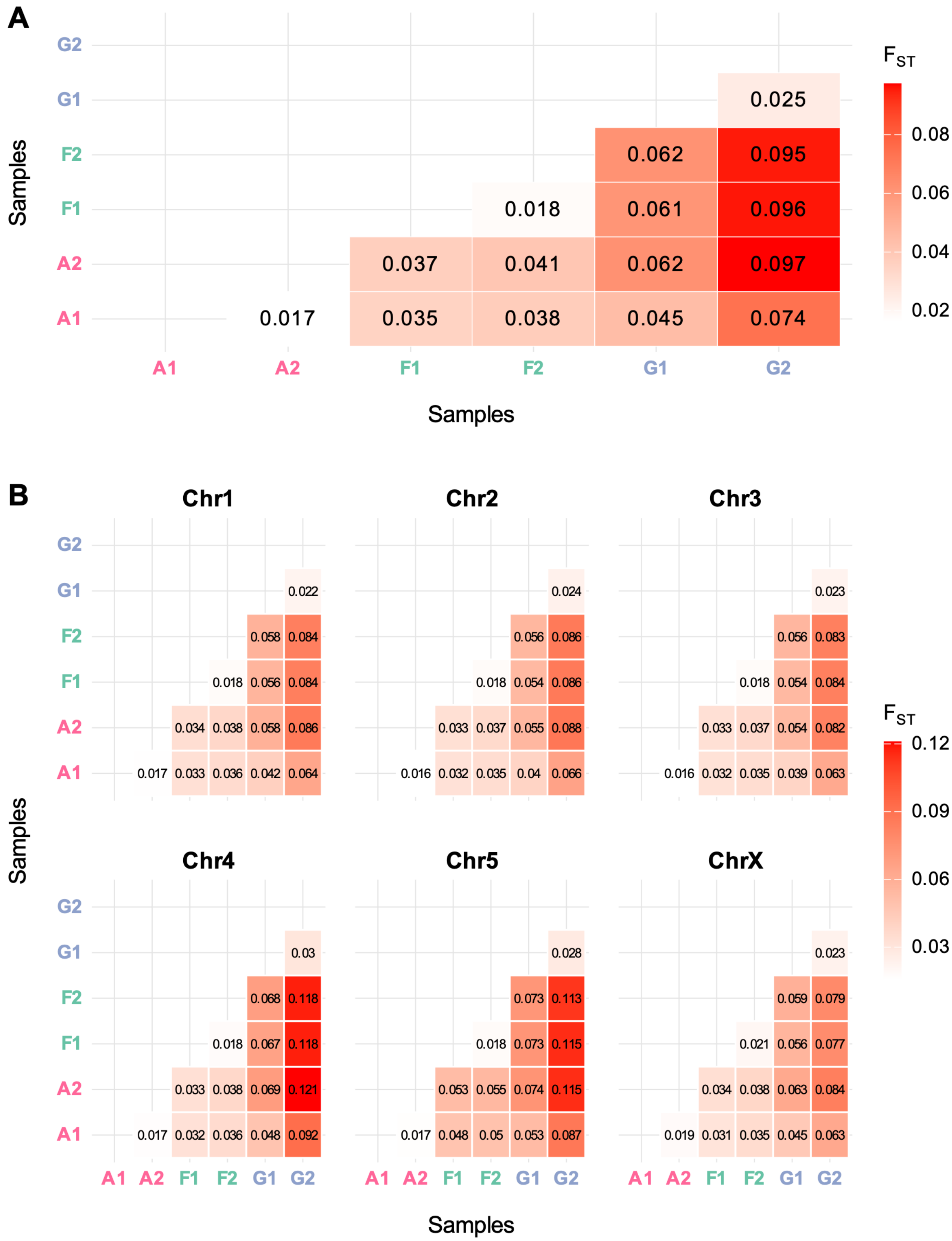

Figure S1. Within-farm genetic differentiation among biological *H. contortus* population replicates (sliding-window F_{ST}, 1 kb window size with 0.5 kb step size). (A) Genome-wide pairwise F_{ST} between replicates from the farms A, F and G. (B) Chromosome-wide pairwise F_{ST} between replicates from the farms A, F and G (chromosomes 1-5 and X). Biological replicates are indicated as follows: samples A1 and A2 from Farm A (pink), samples F1 and F2 from Farm F (green), and samples G1 and G2 from Farm G (purple). Each cell reports an F_{ST} value; The opacity of the red colour cell fill indicates the degree of differentiation.

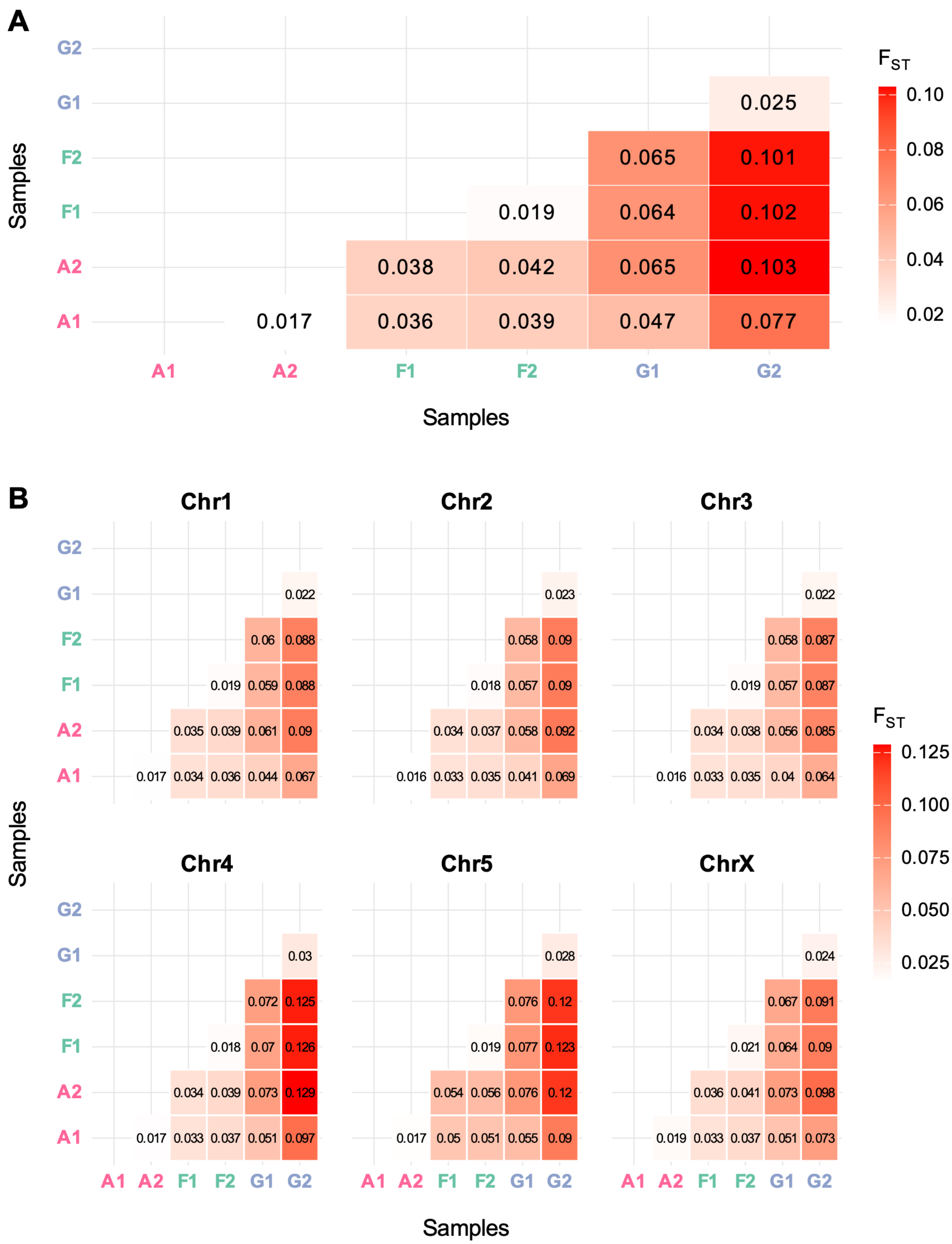
A
B
Samples
FST
Chr1
Chr2
Chr3
Chr4
Chr5
ChrX
A1
A2
F1
F2
G1
G2

Figure S2. Within-farm genetic differentiation among biological *H. contortus* population replicates (sliding-window F_{ST}, 100 kb window size with 50 kb step size). (A) Genome-wide pairwise F_{ST} between replicates from the farms A, F and G. (B) Chromosome-wide pairwise F_{ST} between replicates from the farms A, F and G (chromosomes 1-5 and X). Biological replicates are indicated as follows: samples A1 and A2 from Farm A (pink), samples F1 and F2 from Farm F (green), and samples G1 and G2 from Farm G (purple). Each cell reports an F_{ST} value; The opacity of the red colour cell fill indicates the degree of differentiation.

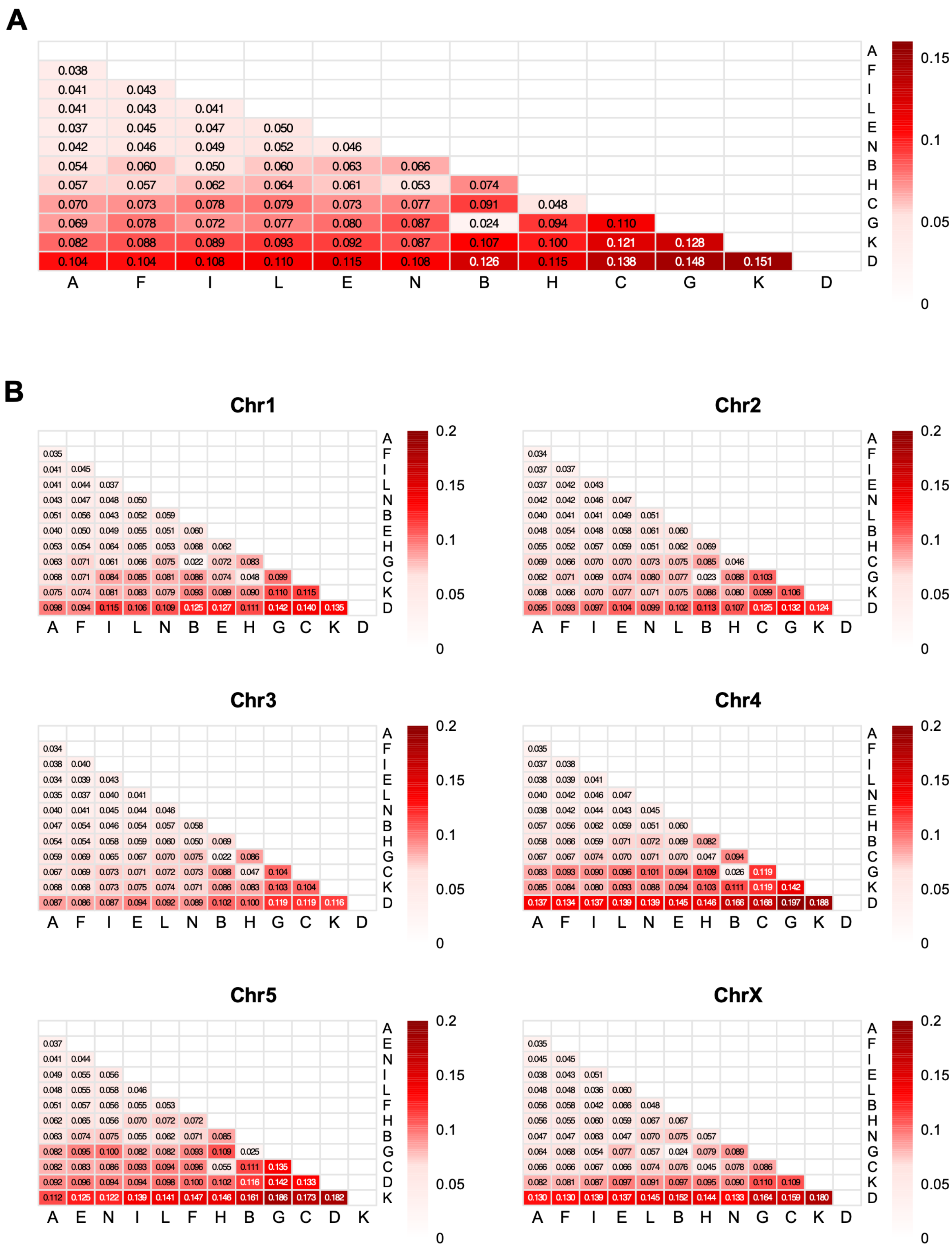

A
0.038
0.041 0.043
0.041 0.043 0.041
0.037 0.045 0.047 0.050
0.042 0.046 0.049 0.052 0.046
0.054 0.060 0.050 0.060 0.063 0.066
0.057 0.057 0.062 0.064 0.061 0.053 0.074
0.070 0.073 0.078 0.079 0.073 0.077 0.091 0.048
0.069 0.078 0.072 0.077 0.080 0.087 0.024 0.094 0.110
0.082 0.088 0.089 0.093 0.092 0.087 0.107 0.100 0.121 0.128
0.104 0.104 0.108 0.110 0.115 0.108 0.126 0.115 0.138 0.148 0.151
A F I L E N B H C G K D
0.15
0.1
0.05
0
B
Chr1
Chr2
Chr3
Chr4
Chr5
ChrX
0.2
0.15
0.1
0.05
0

Figure S3. Between-farm genetic differentiation among *H. contortus* populations (sliding-window F_{ST}, 1 kb window size with 0.5 kb step size). (A) Genome-wide pairwise F_{ST} between *H. contortus* populations from all 12 farms. (B) Chromosome-wide pairwise F_{ST} between *H. contortus* populations from all 12 farms (chromosomes 1-5 and X). For farms A, F, and G, average F_{ST} values of the two biological replicates were used; each cell reports the pairwise F_{ST} value, sorted in an increasing manner, with the opacity of the red colour cell fill indicating the degree of differentiation.

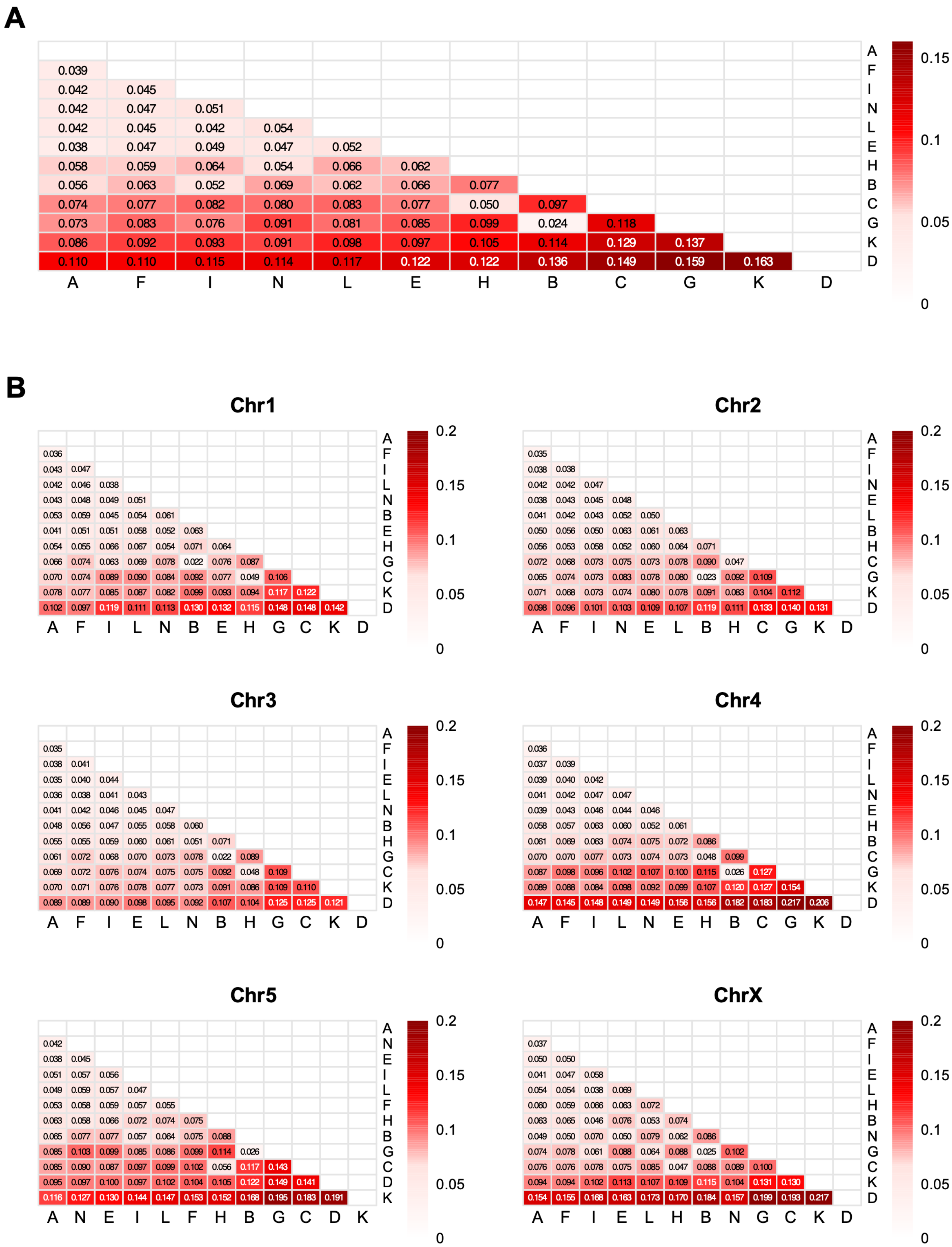
A
A F I N L E H B C G K D
0.15
0.1
0.05
0
0.039
0.042 0.045
0.042 0.047 0.051
0.042 0.045 0.042 0.054
0.038 0.047 0.049 0.047 0.052
0.058 0.059 0.064 0.054 0.066 0.062
0.056 0.063 0.052 0.069 0.062 0.066 0.077
0.074 0.077 0.082 0.080 0.083 0.077 0.050 0.097
0.073 0.083 0.076 0.091 0.081 0.085 0.099 0.024 0.118
0.086 0.092 0.093 0.091 0.098 0.097 0.105 0.114 0.129 0.137
0.110 0.110 0.115 0.114 0.117 0.122 0.122 0.136 0.149 0.159 0.163
B
Chr1
A F I L N B E H G C K D
Chr2
A F I N E L B H C G K D
Chr3
A F I E L N B H G C K D
Chr4
A F I L N E H B C G K D
Chr5
A N E I L F H B G C D K
ChrX
A F I E L H B N G C K D
0.2
0.15
0.1
0.05
0

Figure S4. Between-farm genetic differentiation among *H. contortus* populations (sliding-window F_{ST}, 100 kb window size with 50 kb step size). (A) Genome-wide pairwise F_{ST} between *H. contortus* populations from all 12 farms. (B) Chromosome-wide pairwise F_{ST} between *H. contortus* populations from all 12 farms (chromosomes 1-5 and X). For farms A, F, and G, average F_{ST} values of the two biological replicates were used; each cell reports the pairwise F_{ST} value, sorted in an increasing manner, with the opacity of the red colour cell fill indicating the degree of differentiation.

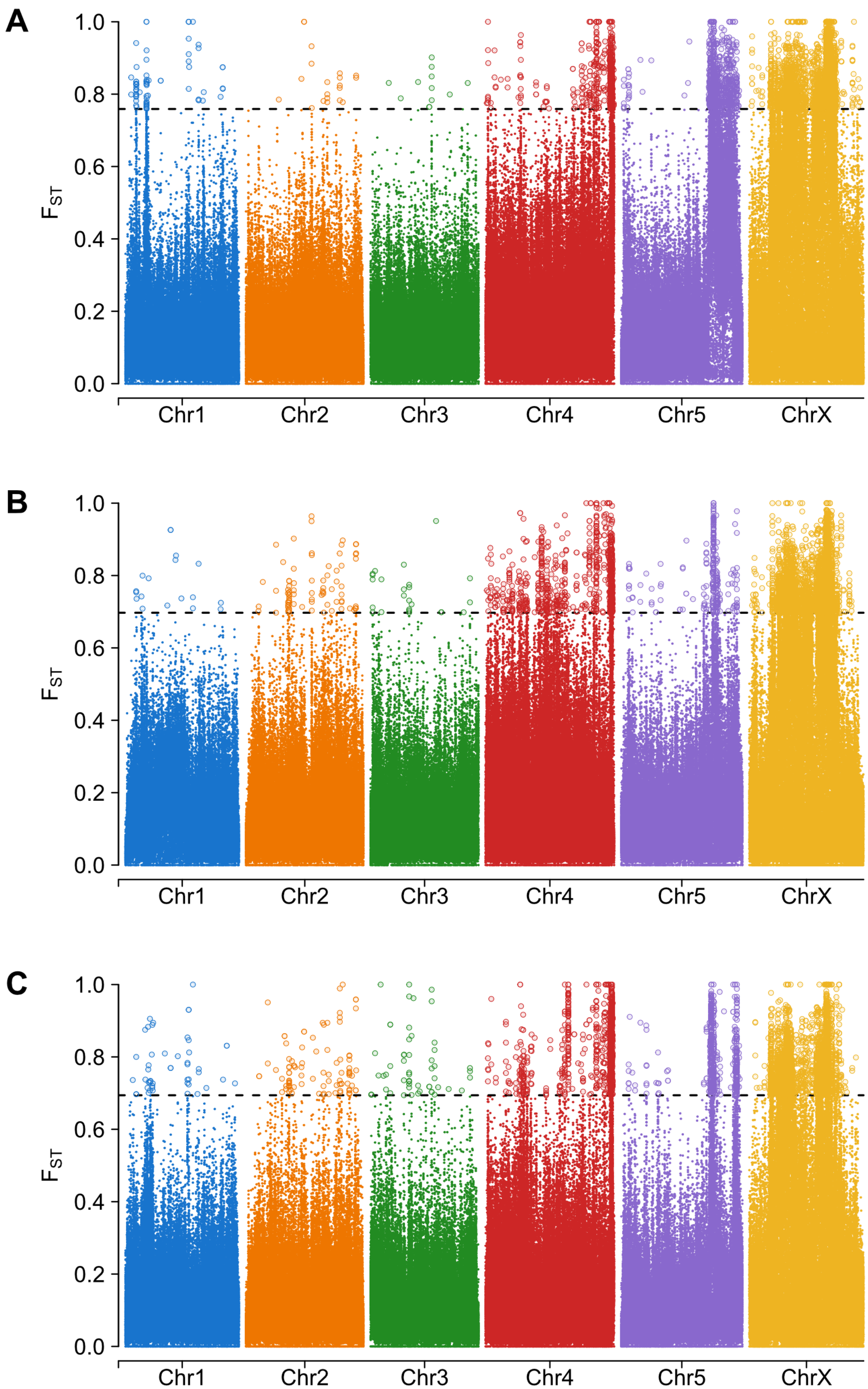
A
B
C
1.0
0.8
0.6
0.4
0.2
0.0
F_{ST}
Chr1
Chr2
Chr3
Chr4
Chr5
ChrX

Figure S5. Sliding-window F_{ST} analysis for the three most divergent *H. contortus* farm population pairs: (A) farms D and K, (B) farms D and G, (C) farms D and C (1 kb window size with 0.5 kb step size). Each dot represents an F_{ST} value calculated for a genomic window along the six *H. contortus* chromosomes. The horizontal dashed lines indicate the top 1% F_{ST} thresholds (A: 0.758, B: 0.697, C: 0.694), and windows exceeding these thresholds are highlighted with larger open circles.

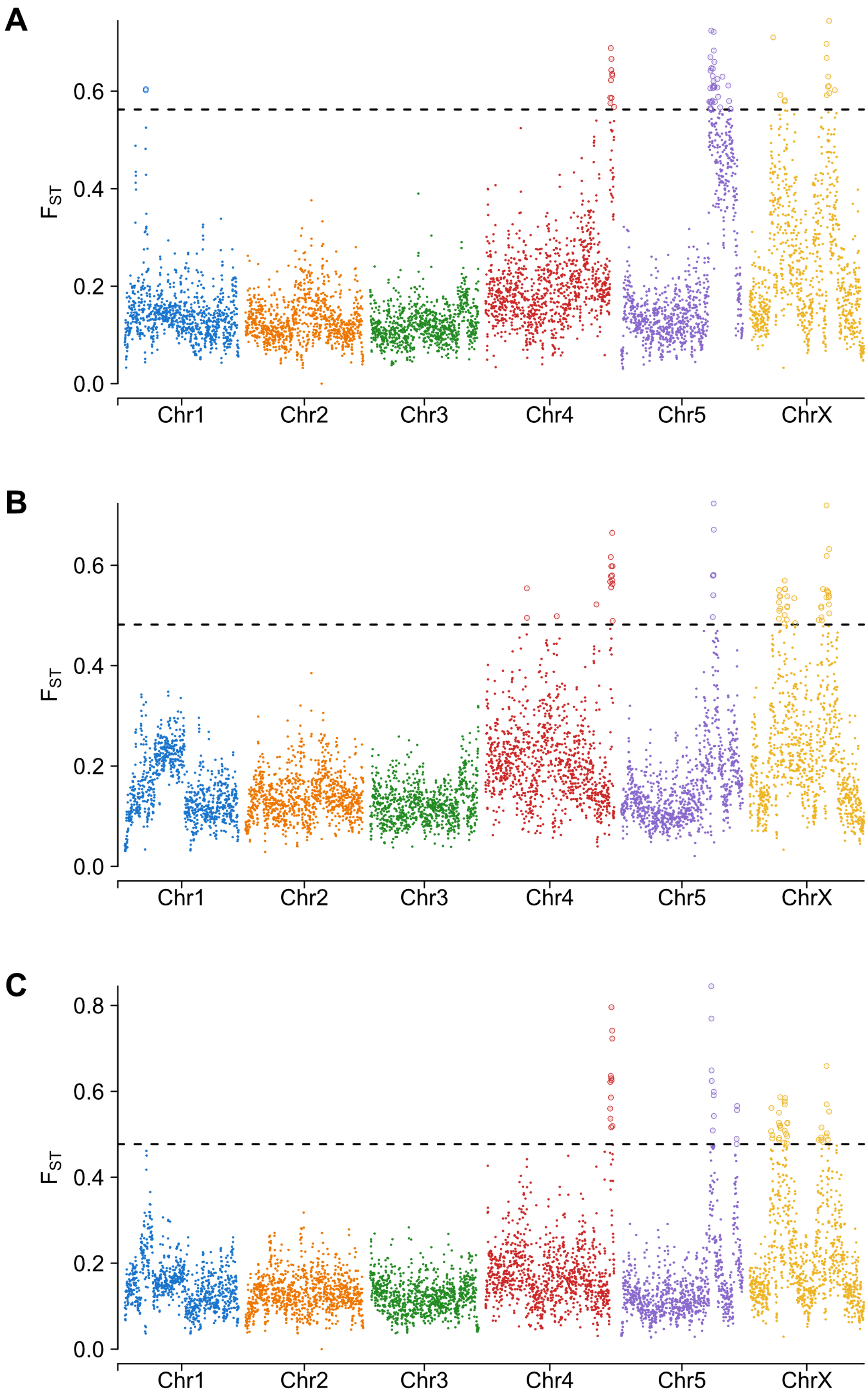

A
B
C
F_ST
0.0
0.2
0.4
0.6
0.8
Chr1
Chr2
Chr3
Chr4
Chr5
ChrX

Figure S6. Sliding-window F_{ST} analysis for the three most divergent *H. contortus* farm population pairs: (A) farms D and K, (B) farms D and G, (C) farms D and C (100 kb window size with 50 kb step size). Each dot represents an F_{ST} value calculated for a genomic window along the six *H. contortus* chromosomes. The horizontal dashed lines indicate the top 1% F_{ST} thresholds (A: 0.562, B: 0.481, C: 0.477), and windows exceeding these thresholds are highlighted with larger open circles.

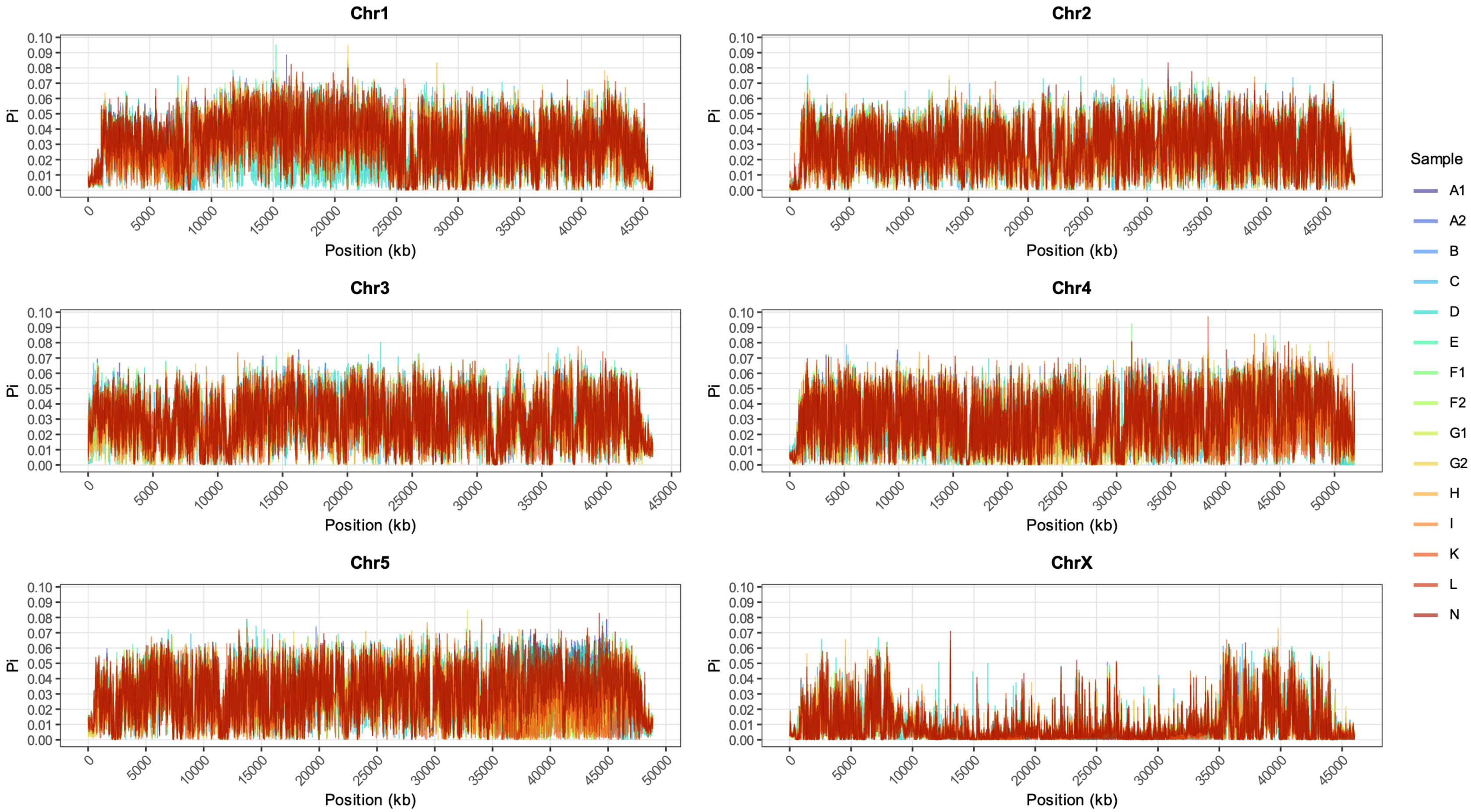

Figure S7. Chromosome-wide landscapes of window-based nucleotide diversity (Pi) estimates within the 15 *H. contortus* population samples from the 12 farms (Farms A, F, and G, each contributed two biological replicates). Window-based Pi estimates are shown in 10 kb windows along chromosomes 1–5 and X, with the x-axis indicating chromosomal position (kb).

	F	B	L	C	H	I	A	E	N	D	G	K
F												
B	7.943e-07											
L	1.225e-03	3.766e-15										
C	2.419e-21	7.839e-06	1.706e-36									
H	5.680e-14	4.811e-33	2.422e-06	2.836e-61								
I	2.689e-13	8.487e-33	1.269e-05	1.264e-61	7.139e-01							
A	1.923e-14	2.107e-34	1.883e-06	1.264e-63	2.906e-01	2.823e-01						
E	6.539e-20	1.260e-42	3.790e-10	1.967e-74	1.286e-01	5.689e-02	6.525e-02					
N	8.194e-41	8.280e-70	3.065e-27	7.990e-109	3.736e-09	2.029e-10	6.477e-10	1.186e-05				
D	4.997e-59	2.481e-29	7.009e-84	3.364e-12	2.891e-116	1.956e-118	6.968e-121	9.672e-135	7.656e-175			
G	9.079e-67	9.209e-37	1.605e-92	3.907e-20	6.336e-125	4.771e-127	9.492e-130	1.357e-143	2.017e-185	2.120e-11		
K	3.966e-80	1.276e-44	7.990e-109	2.207e-22	3.735e-146	5.207e-148	1.234e-150	2.441e-166	2.012e-211	6.457e-03	6.971e-14	

200 150 100 50

Figure S8. Pairwise comparisons of genome-wide, window-based nucleotide diversity (Pi) distributions between the 12 *H. contortus* farm populations. Each cell reports the Benjamini-Hochberg-adjusted P-value from a pairwise Wilcoxon Rank Sum test. Darker purple shading indicates smaller adjusted P-values and stronger statistical significance. The colour scale on the right is based on the absolute value of the exponent in scientific notation. For farms A, F and G, P-values from two replicates were combined using the geometric mean.
